# Supplementary material for: NLRP3 lacking the leucine-rich repeat domain can be fully activated via the canonical inflammasome pathway
Source: Nat Commun. 2018 Dec 5;9:5182. doi: 10.1038/s41467-018-07573-4 (PMC6281599; doi:10.1038/s41467-018-07573-4)
Supplement: Supplementary file 1 — Supplementary Information [file 41467_2018_7573_MOESM1_ESM.pdf]

Supplementary Information

NLRP3 lacking the leucine-rich repeat domain can be fully activated via the canonical inflammasome pathway

Hafner-Bratkovič et al.

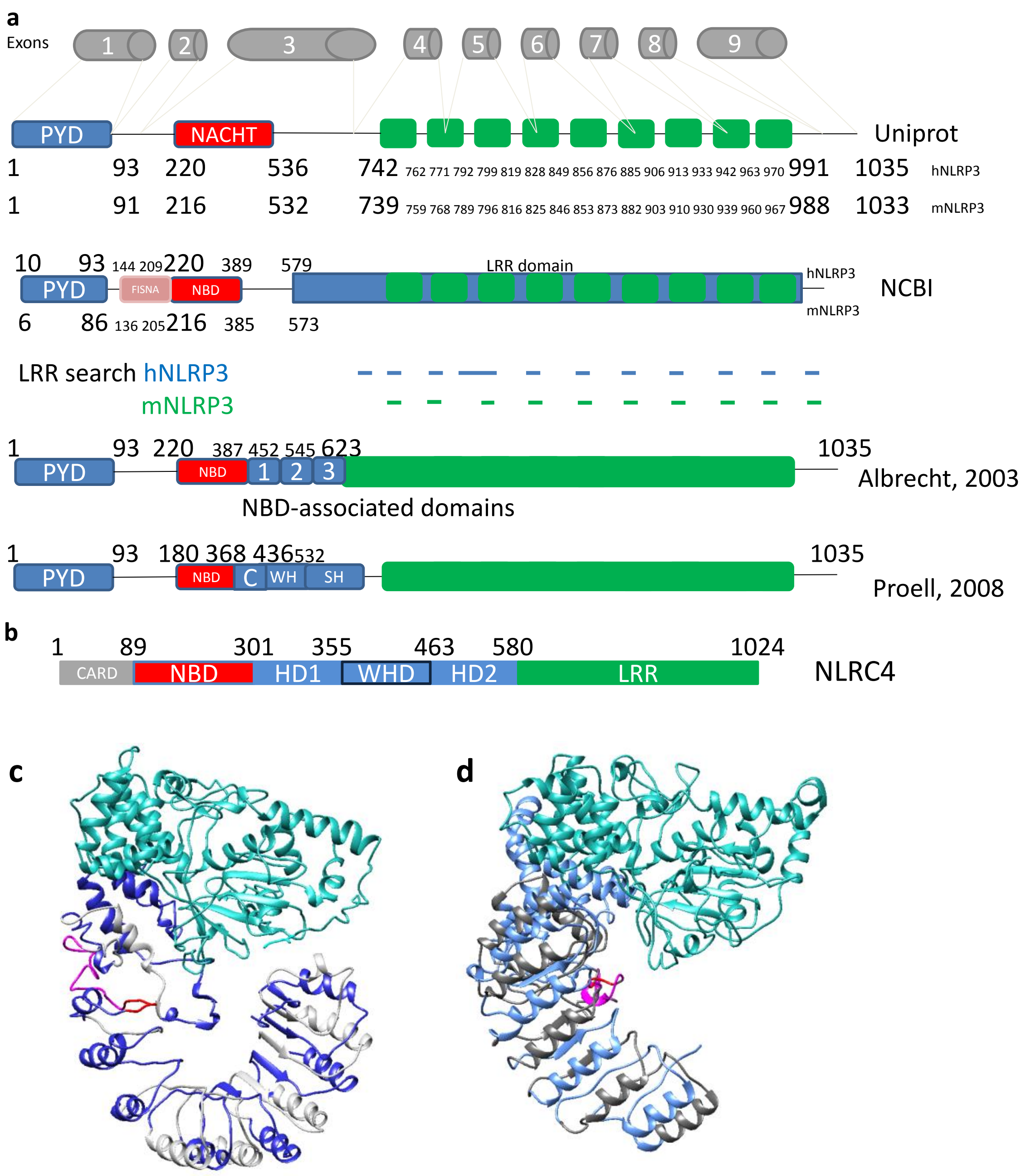

Supplementary Fig. 1. Annotations and models of NLRP3. **a** Truncation variants were designed based on annotations from Uniprot[1] and NCBI, LRR prediction tool LRRsearch[2] and published studies[3, 4]. Note that NCBI annotation predicts a long LRR domain starting at amino acid residue 579, as opposed to the majority of others (LRR domain starts after 700). NACHT domain is also differently annotated. NCBI and published studies[3, 4] use it synonymously to nucleotide-binding domain (NBD). NCBI predicts N-terminal FISNA (Fish-specific NACHT associated domain) **b** Domain representation of NLRC4. **c,d** Homology models of mouse NLRP3 based on the structures of NLRC4 (c)[5] and NLRC2 (d) [6] were predicted by I-TASSER [7]. Iterative blue and gray colors depict the borders of designed truncated variants. The region between 665 and 686 is depicted in pink. Abbreviations used: C-helical domain; WH, WHD-winged helical domain; SH-superhelical domain; HD1-helical domain 1; HD2-helical domain 2.

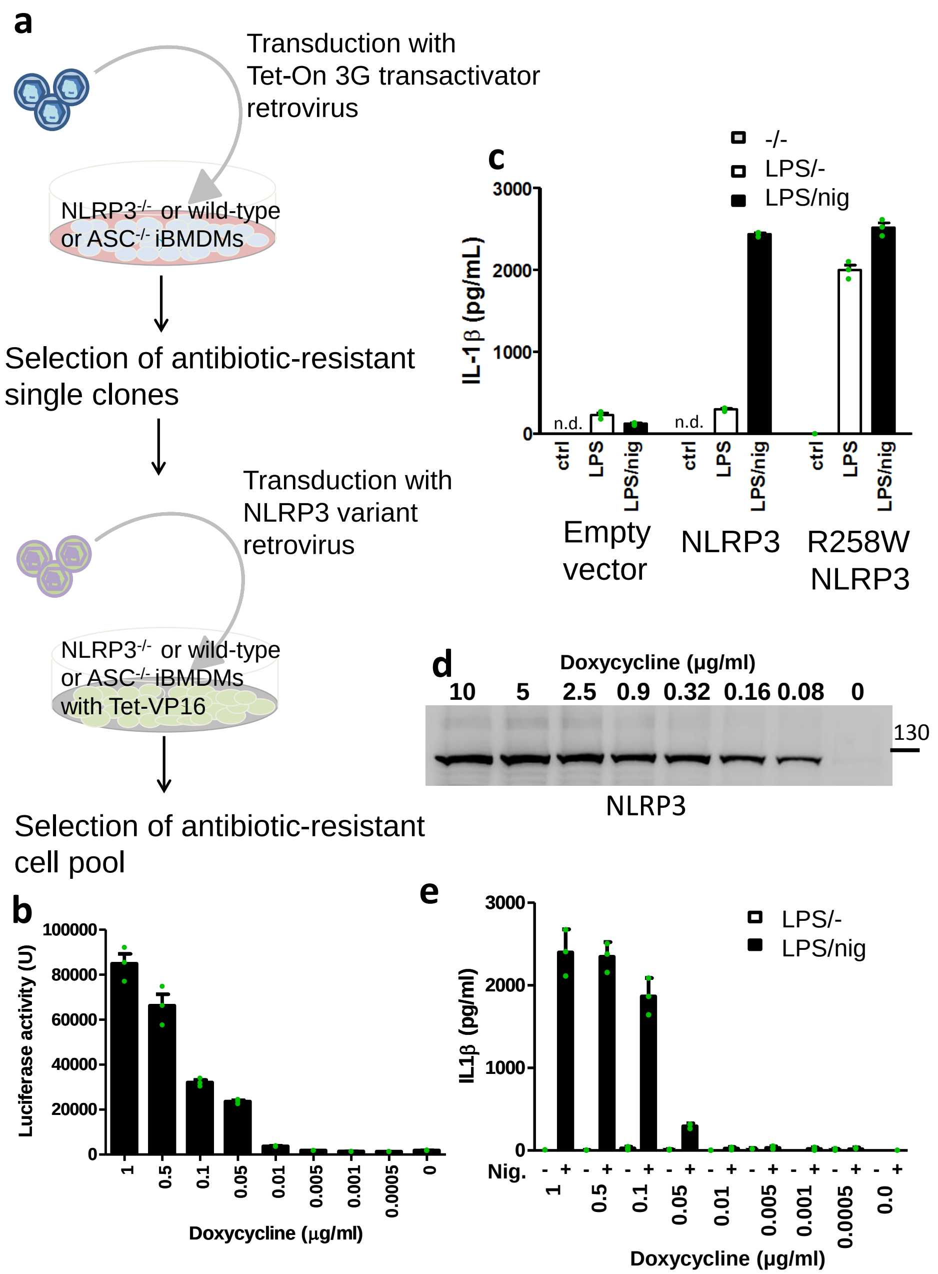

Supplementary Fig. 2. Validation of the inflammasome reconstitution system. **a** NLRP3$^{-/-}$, ASC$^{-/-}$ or wild-type iBMDMs were first transduced with retrovirus encoding Tet-ON transactivator and later with retrovirus encoding NLRP3 variants or other innate immune genes. **b** The system was first validated in macrophages transduced with control (firefly luciferase gene) by doxycycline titration. **c**) NLRP3$^{-/-}$ iBMDMs were transduced with empty vector, wild-type NLRP3 or NLRP3 R258W and tested for activation with nigericin and constitutive activation. Secretion of IL-1β from non-treated cells was below detection limit (n.d.). **d,e** Doxycycline titration of NLRP3$^{-/-}$ iBMDMs reconstituted with wild-type mouse NLRP3 was performed to follow protein expression (**d**) and nigericin activation (**e**). Representative of 2 (**b, d, e**) or 5 (**c**) experiments is shown, the mean and the s.e.m. of 2 or 3 biological replicates are shown (**b, c, e**).

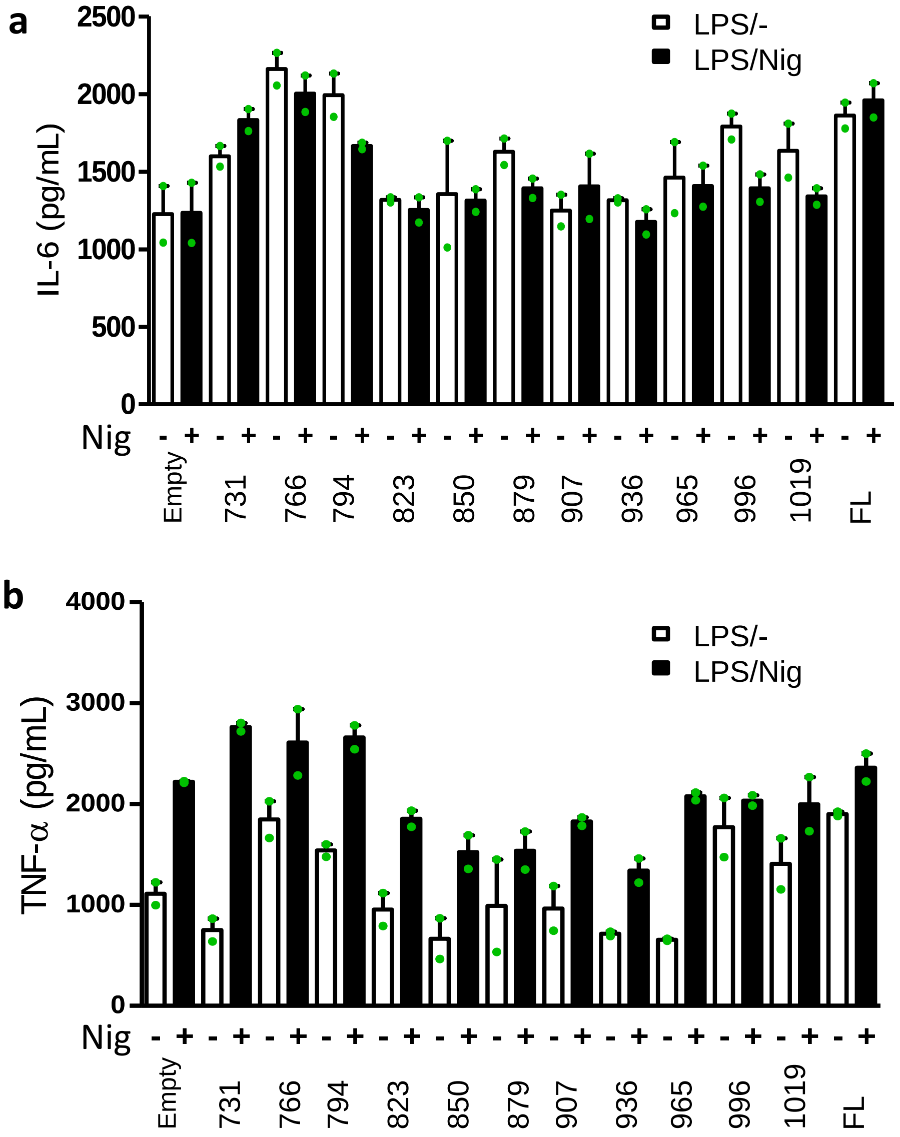

Supplementary Fig. 3. IL-6 (**a**) and TNF-α (b) levels released from stimulated NLRP3$^{-/-}$ iBMDMs. Immortalized mouse bone-marrow derived macrophages from NLRP3$^{-/-}$ mice with stable integration of designated constructs were primed with LPS (100 ng/ml) and doxycycline (0.05 μg/ml) for 11 h. After priming, the medium was exchanged and cells were stimulated with nigericin (5 μM) for 1 h. Representative of 2 independent experiments is shown. The mean and the s.e.m. of 2 biological replicates (**a,b**) are shown.

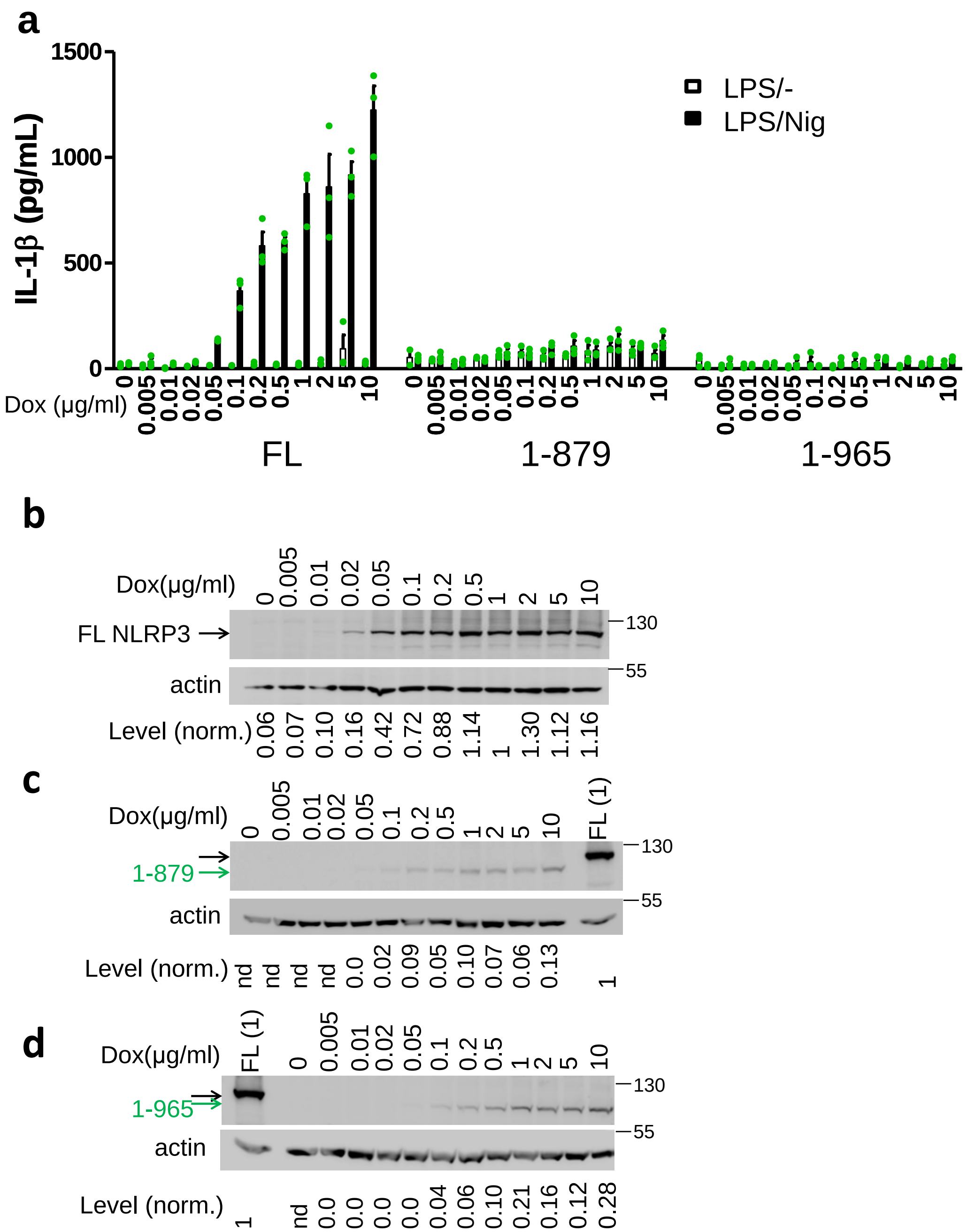

Supplementary Fig. 4. Doxycycline titration of selected NLRP3$^{-/-}$ iBMDMs. Cells were primed with LPS (100 ng/mL) and increasing concentrations of doxycycline (in µg/ml) for 12 h, after which the medium was exchanged and cells were stimulated with nigericin (5 µM, 1 h) (**a**). Protein levels of FL (**b**), 1-879 (**c**) and 1-965 (**d**) after priming (LPS + doxycycline) were first normalized to loading controls (actin) and afterwards to normalized FL (1) value (protein level of FL induced by 1 µg/ml doxycycline) on the same blot. Representative of 2 independent experiments is shown (**a-d**). The mean and the s.e.m. of 3 biological replicates are shown (**a**).

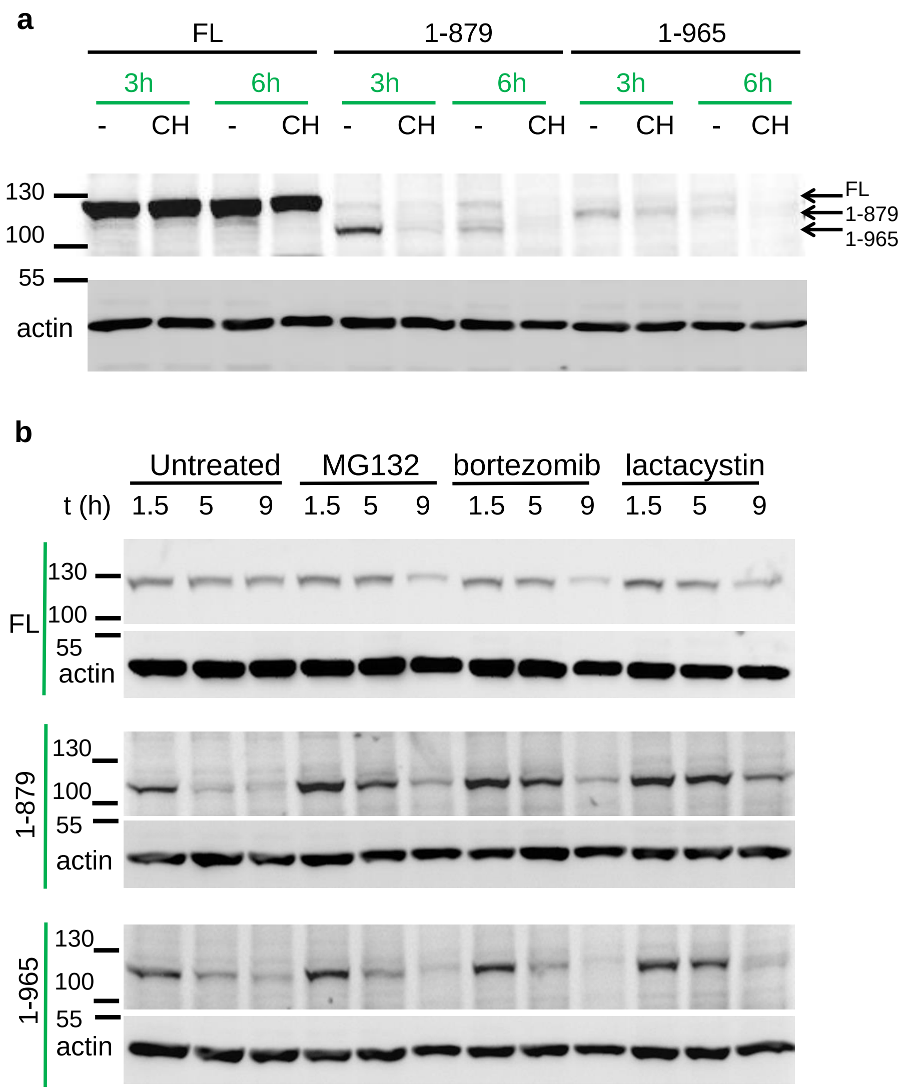

Supplementary Fig. 5. Stability of full-length (FL), 1-879 and 1-965 mNLRP3 variants. iBMDMs reconstituted with FL mNLRP3, 1-879 and 1-965 variants were incubated in DMEM supplemented with FBS and 0.1 µg/ml doxycycline for 11 h, after which medium was replaced with FBS supplemented DMEM with cycloheximide (CH) (100 µM) (**a**) or proteasome inhibitors MG132 (20 µM), bortezomib (10 µM) and lactacystin (50 µM) (**b**) for the defined time, after which cells were lysed and protein levels were followed by Western blot. Representative of 2 independent experiments is shown (**a,b**). Note that 9 h incubation with proteasome inhibitors affected cell viability, which is also observed by the loss of FL protein expression.

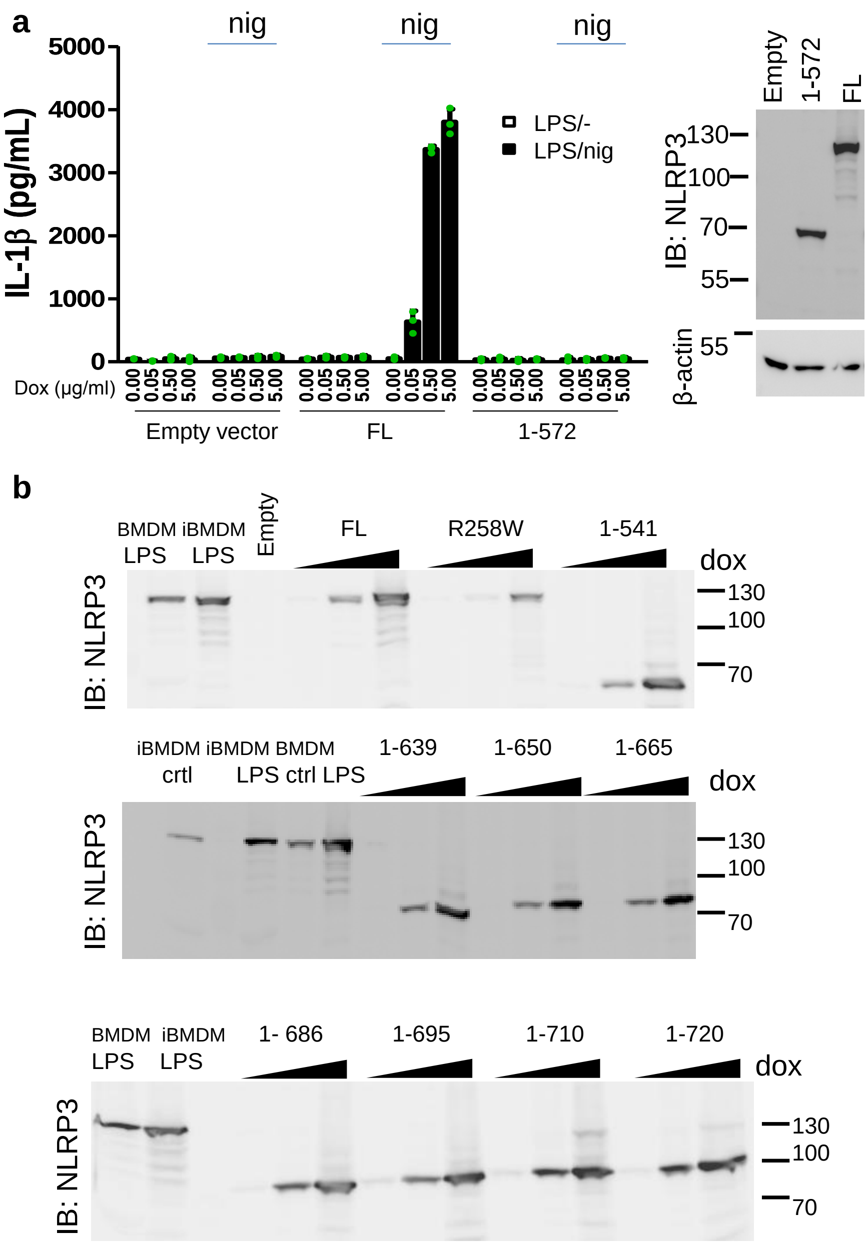

Supplementary Fig. 6. Comparable expression of NLRP3 variants and endogenous NLRP3. **a** Although 1-572 is expressed comparably to FL, it does not support inflammasome activation in response to nigericin. Cells were primed with LPS (100 ng/ml) and doxycycline (various concentrations) for 10 h and afterwards treated with nigericin (5 μM) for 1h. **b** Expression levels of truncated NLRP3 variants upon induction with 0, 0.5 and 2 μg/ml doxycycline. Expression of endogenous NLRP3 in primary or immortalized BMDMs was induced by LPS (100 ng/ml for 6 h). Representative of 3 (**a**) or 2 (**b**) independent experiments is shown. The mean and the SEM of three biological replicates are shown (**a**).

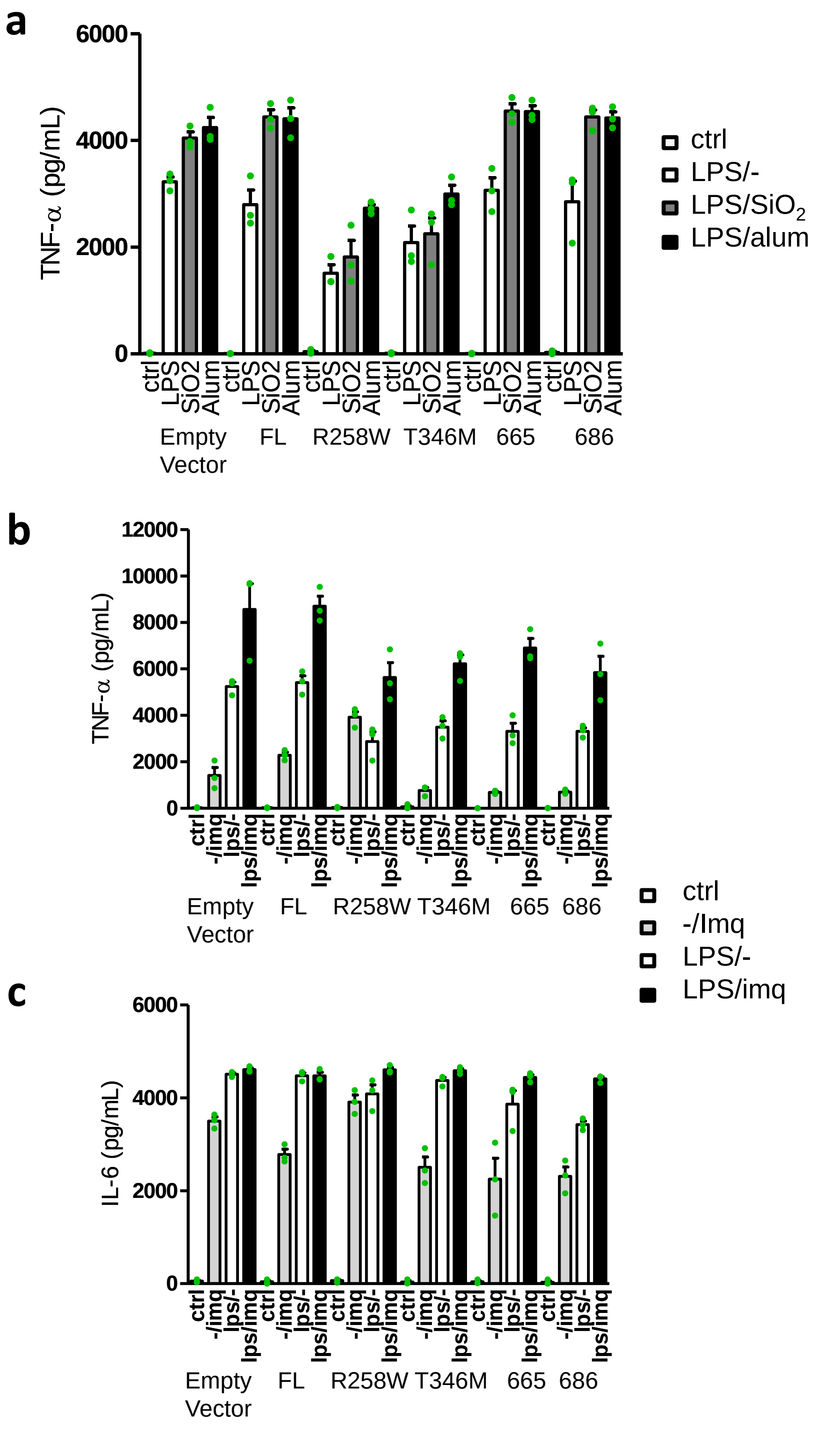

Supplementary Fig. 7. IL-6 and TNF-α levels released from stimulated NLRP3$^{-/-}$ iBMDMs. After priming and stimulation (**a**) supernatants from Fig. 2e were analyzed for TNF-α secretion and (**b,c**) supernatants from Fig. 2f were analyzed for TNF-α and IL-6 release. Representative of 2 independent experiments is shown (**a-c**). The mean and the s.e.m. of 3 biological replicates are shown (**a-c**).

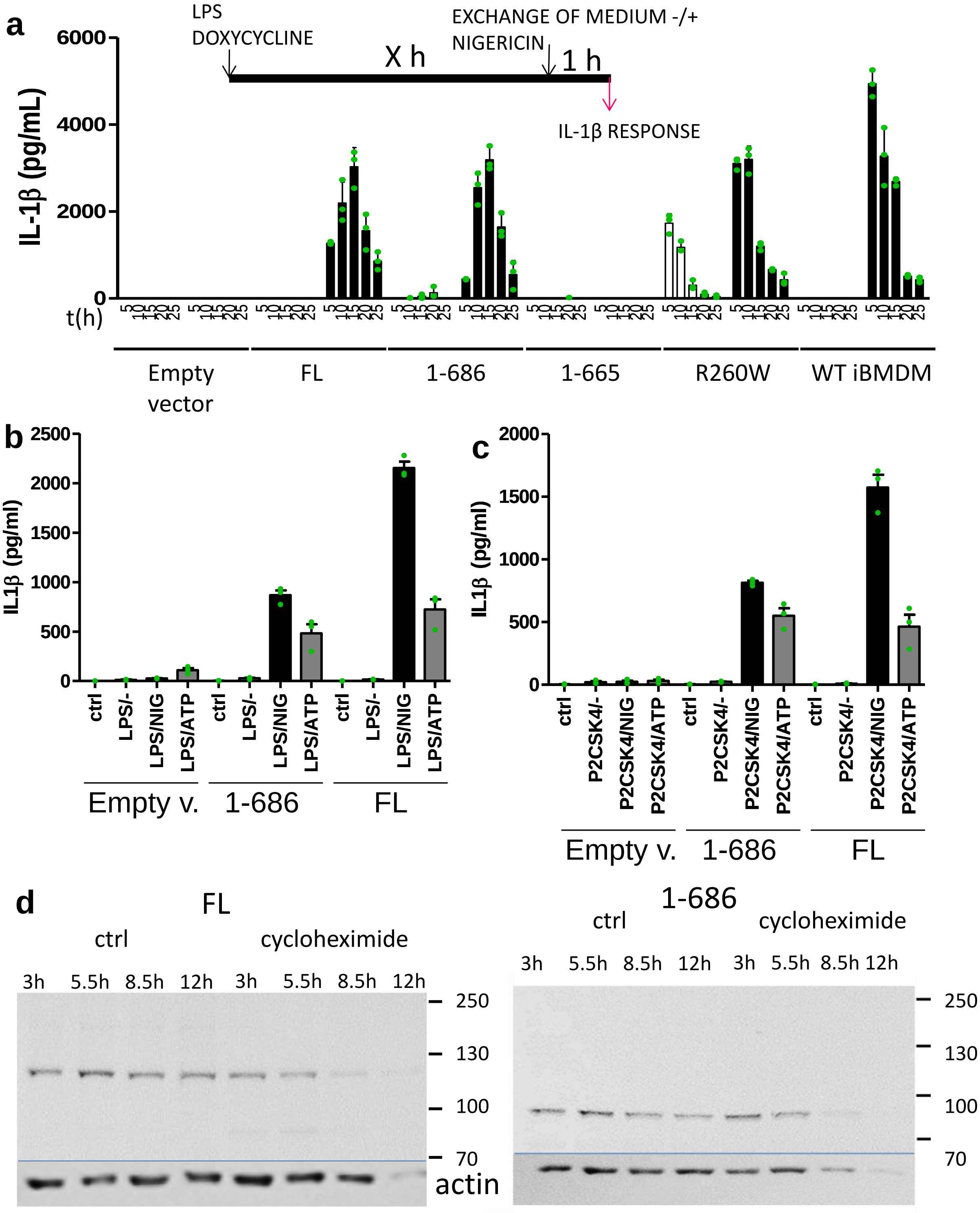
a
IL-1β (pg/mL)
6000
4000
2000
0
LPS
DOXYCYCLINE
X h
EXCHANGE OF MEDIUM -/+
NIGERICIN
1 h
IL-1β RESPONSE
t(h)
Empty vector
FL
1-686
1-665
R260W
WT iBMDM
b
IL1β (pg/ml)
ctrl
LPS/-
LPS/NIG
LPS/ATP
Empty v.
1-686
FL
c
P2CSK4/-
P2CSK4/NIG
P2CSK4/ATP
d
FL
1-686
ctrl
cycloheximide
3h
5.5h
8.5h
12h
250
130
100
70
actin

e

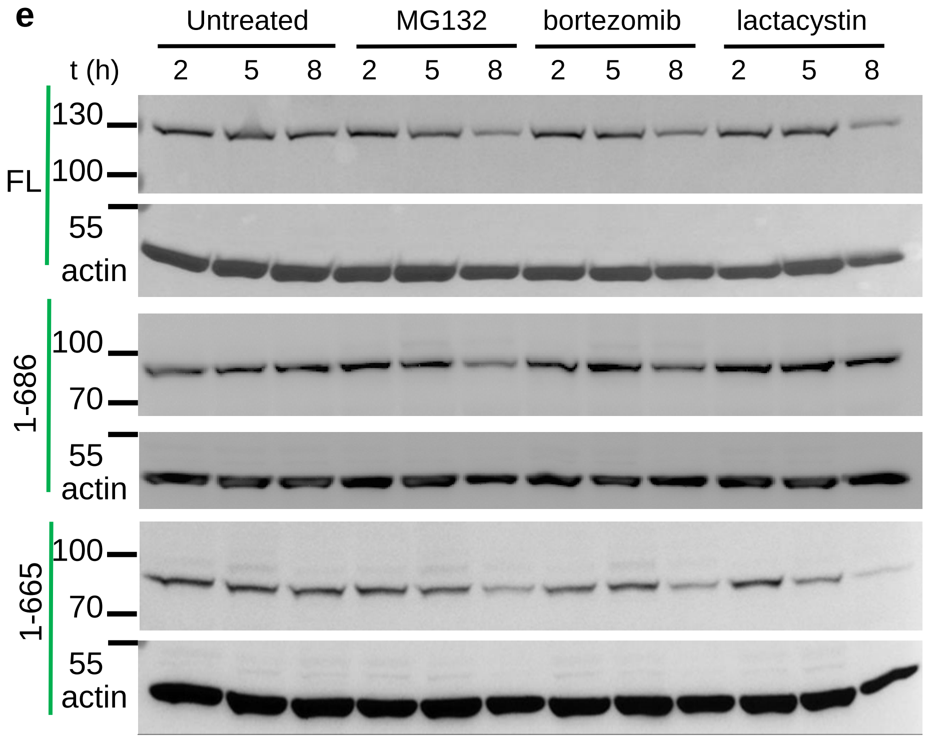

Supplementary Fig. 8. MiniNLRP3 (1-686) has similar phenotype as full length NLRP3.
a Stable NLRP3$^{-/-}$ iBMDMs harboring different NLRP3 variants and wild-type iBMDMs were primed with LPS and doxycycline for various duration, after which the media was replaced, and nigericin (5 µM) was added for 1 h, after which IL-1β maturation was followed. Where no data points are shown, IL-1β was below detection limit. **b,c** Cells were first incubated in DMEM with doxycycline (0.5 µg/ml) for 12 h, after which LPS (100 ng/ml) (**b**) or synthetic diacylated lipopeptide P2CSK4 (1 µg/ml) (**c**) were added for 5h. After priming period medium was exchanged and cells were stimulated with nigericin (5 µM) or ATP (5 mM) for 45 min.
d,e NLRP3$^{-/-}$ iBMDMs reconstituted with either FL or 1-686 variant were primed for 12 h, after which the media was exchanged and cells were treated with (**d**) cycloheximide (100 µg/ml) or (**e**) proteasome inhibitors MG132 (20 µM), bortezomib (10 µM) and lactacystin (50 µM) for designated time. Note that 8 h incubation with proteasome inhibitors affected cell viability, which is also observed by the loss of FL protein expression. Representative of two independent experiments is shown (**a-e**). The mean and the s.e.m. of three biological replicates are shown (**a,b,c**).

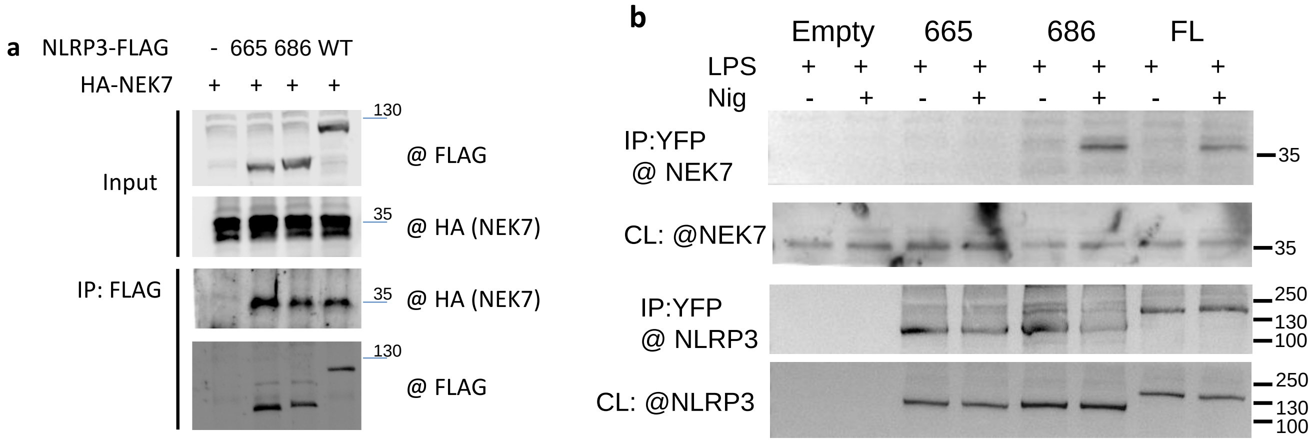

Supplementary Fig. 9. MiniNLRP3 (1-686) interacts with NEK7 kinase. **a** Interaction of HA-NEK7 and FLAG-tagged mNLRP3 variants was followed by immunoprecipitation. Plasmids encoding HA-NEK7 and NLRP3-FLAG variants were co-transfected into HEK293 cells. Upon lysis, FLAG-tagged proteins were pulled down by magnetic beads and protein content was analyzed for the presence of NEK7. **b** NLRP3$^{-/-}$ iBMDMs with stably integrated NLRP3-YFP-tagged variants were primed with LPS (100 ng/ml) and doxycycline (0.5 µg/ml) for 12 h prior to Z-VAD-FMK (10 µg/ml) incubation and nigericin activation (5 µM, 30 min). Upon lysis, NLRP3 variants were pulled down with anti-GFP antibody and protein content was analyzed for the presence of NEK7. Representative of 2 experiments is shown.

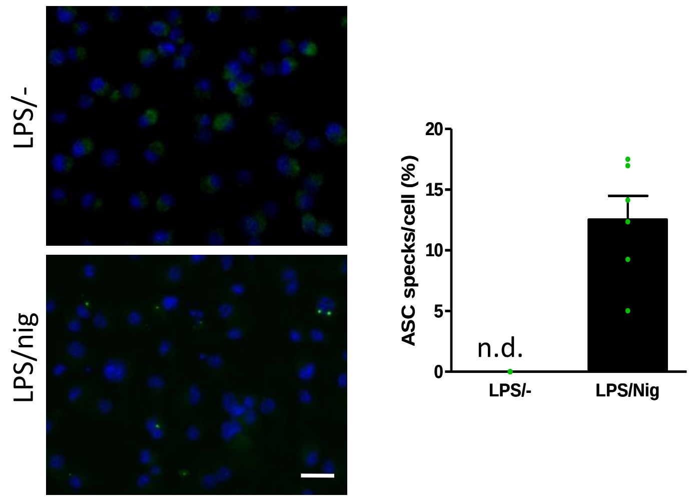

Supplementary Fig. 10. ASC speck formation in iBMDMs from wild-type mice**.** LPS-primed and nigericin-treated immortalized BMDMs (WT iBMDM) were analyzed for ASC speck formation. Nuclei are depicted in blue (DAPI) and ASC in green; bar represents 20 µm. No specks were detected in five random 138 X 110 µm^2 frames for primed-only cells. ASC specks per cell were analyzed in six random frames. Representative of 2 independent experiments is shown.

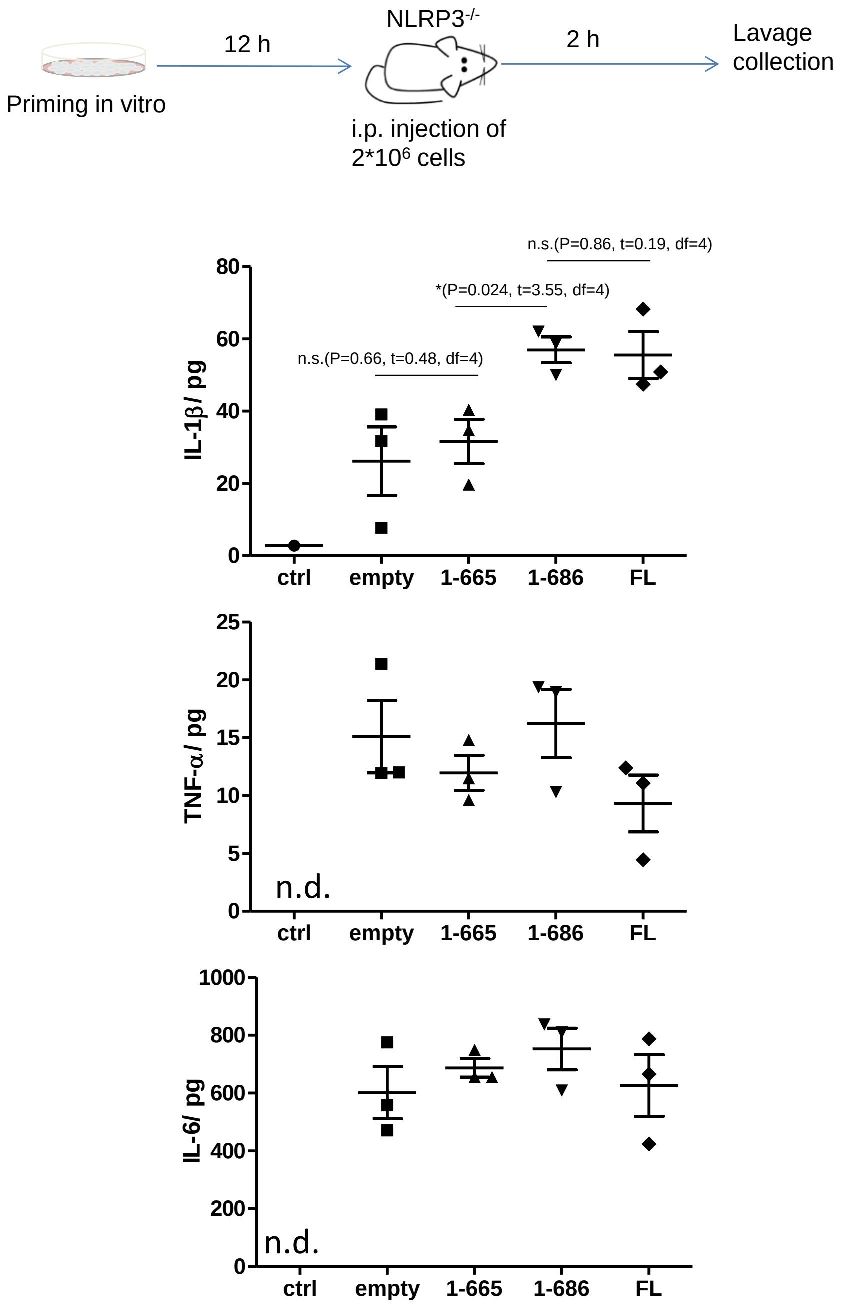

Supplementary Fig. 11. Cytokine levels in peritoneal lavage in mouse peritonitis model. Primed and doxycycline-stimulated NLRP3$^{-/-}$ iBMDMs carrying either the empty vector, 1-665, 1-686 (MiniNLRP3), or the full-length mouse NLRP3 were injected into the peritoneal cavity of NLRP3$^{-/-}$ mice, and cytokine levels in peritoneal lavage were analyzed 2 h after injection. 3 animals were used per each condition. The mean and the s.e.m. are depicted. Statistical values: n.s. P>0.05, * P<0.05. The two-tailed nonparametric t-test was used for pairwise comparison.

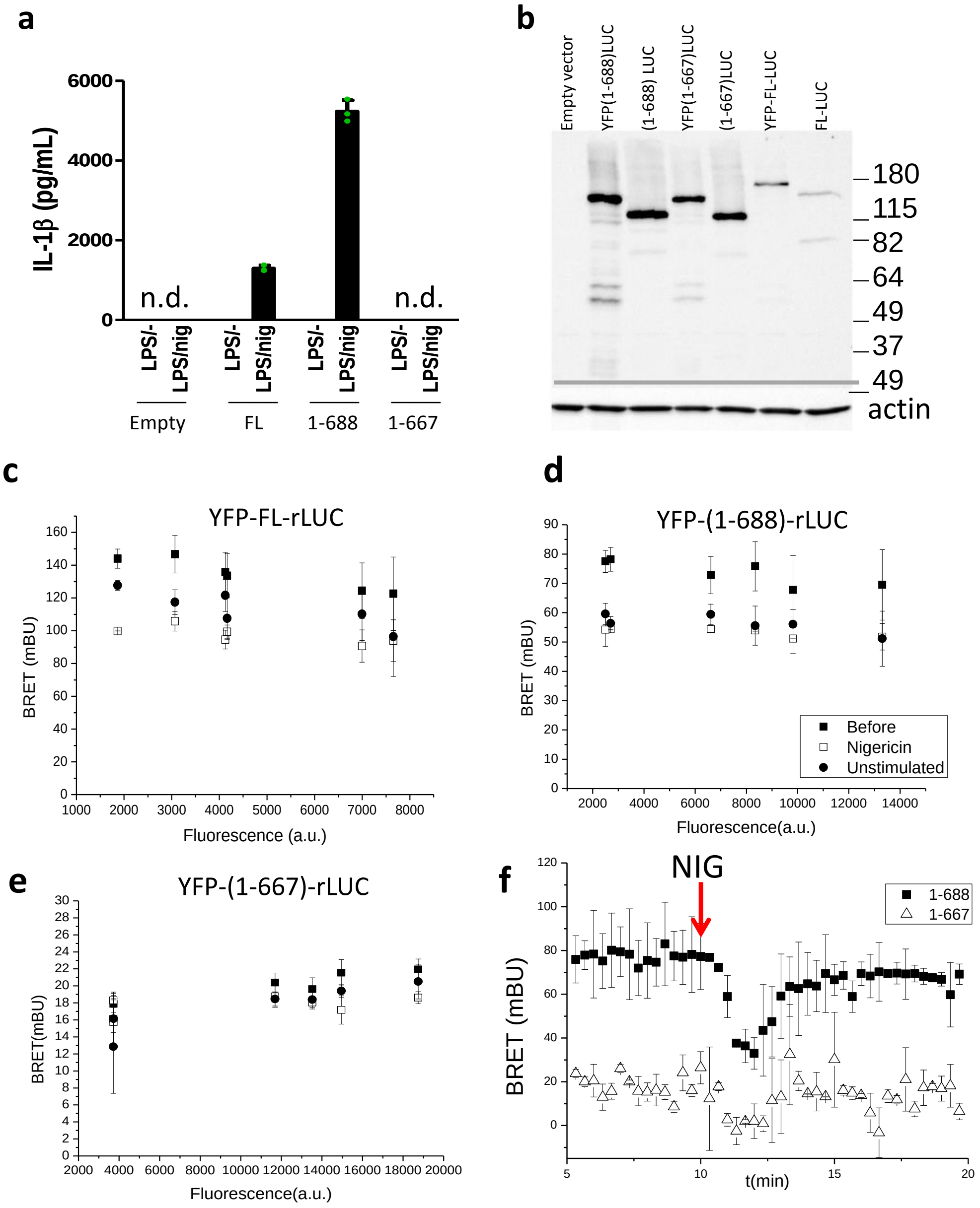

Supplementary Fig. 12. Intramolecular BRET studies of human NLRP3 variants. **a** N-terminally YFP- and C-terminally rLUC-tagged full length and miniNLRP3 (1-688) retained the ability to process IL-1β in response to nigericin. NLRP3$^{-/-}$ iBMDMs with stably integrated double tagged NLRP3 variants were primed with LPS (100 ng/ml) and doxycycline (1 µg/ml) for 11 h prior to nigericin activation (10 µM, 1h). **b** Expression of double-tagged NLRP3 variants in NLRP3$^{-/-}$ iBMDMs primed as in (**a**). **c-e** BRET titration experiment of double-tagged NLRP3 variants in HEK 293 cells shows no concentration dependence indicating that the BRET signal observed originates primarily from intramolecular excitation. Average resting (■, prior to injection of buffer or nigericin) and steady-state nigericin (□) and buffer (●) stimulated signals are depicted. **f** Intramolecular BRET of miniNLRP3 and 1-667 followed in iBMDMs primed with 100 ng/ml LPS and 5 µg/ml doxycycline for 12 h prior to BRET measurement. Representative of 3 (**a,b,f**) or 2(**c-e**) experiments is shown. The mean and the s.d. of 3 (**a**) or 2 (**c-f**) biological replicates are shown.

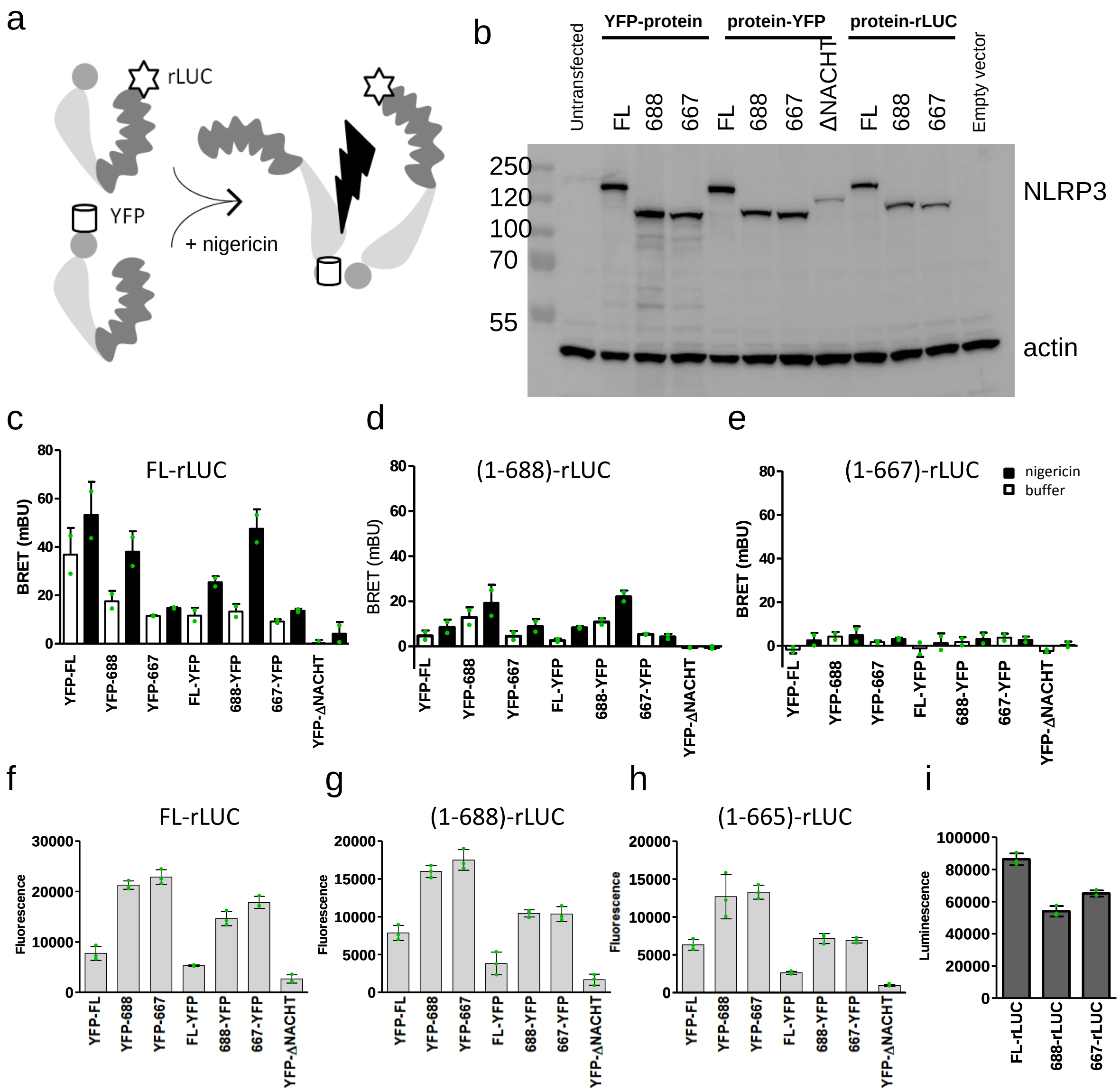

Supplementary Fig. 13. Intermolecular BRET studies of human NLRP3 variants. **a** Intermolecular BRET is observed when rLUC donor on one molecule is in proximity of YFP acceptor on the second molecule. **b** YFP- and rLUC NLRP3 variants expressed in HEK293 cells. **c-e** Steady-state intermolecular BRET followed after transfection of combinations of C-terminally rLUC-tagged NLRP3 variants with indicated acceptor variants (YFP-protein: protein with YFP on the N-terminus; protein-YFP: protein with YFP on the C-terminus) after nigericin or buffer stimulation. **f-h** To perform BRET studies, transfected cells are plated into separate plates for luminescence and fluorescence readings. Background reduced fluorescence intensity correlates to acceptor expression. **i** Basal luminescence of donor-only transfected constructs (485 nm) correlates to donor construct expression. Representative of 2 experiments is shown (**b-i**). The mean and the s.d. of 2 (**c-e**) or 3 (**f-i**) biological replicates are shown. MiniNLRP3 is represented by human 1-688 variants.

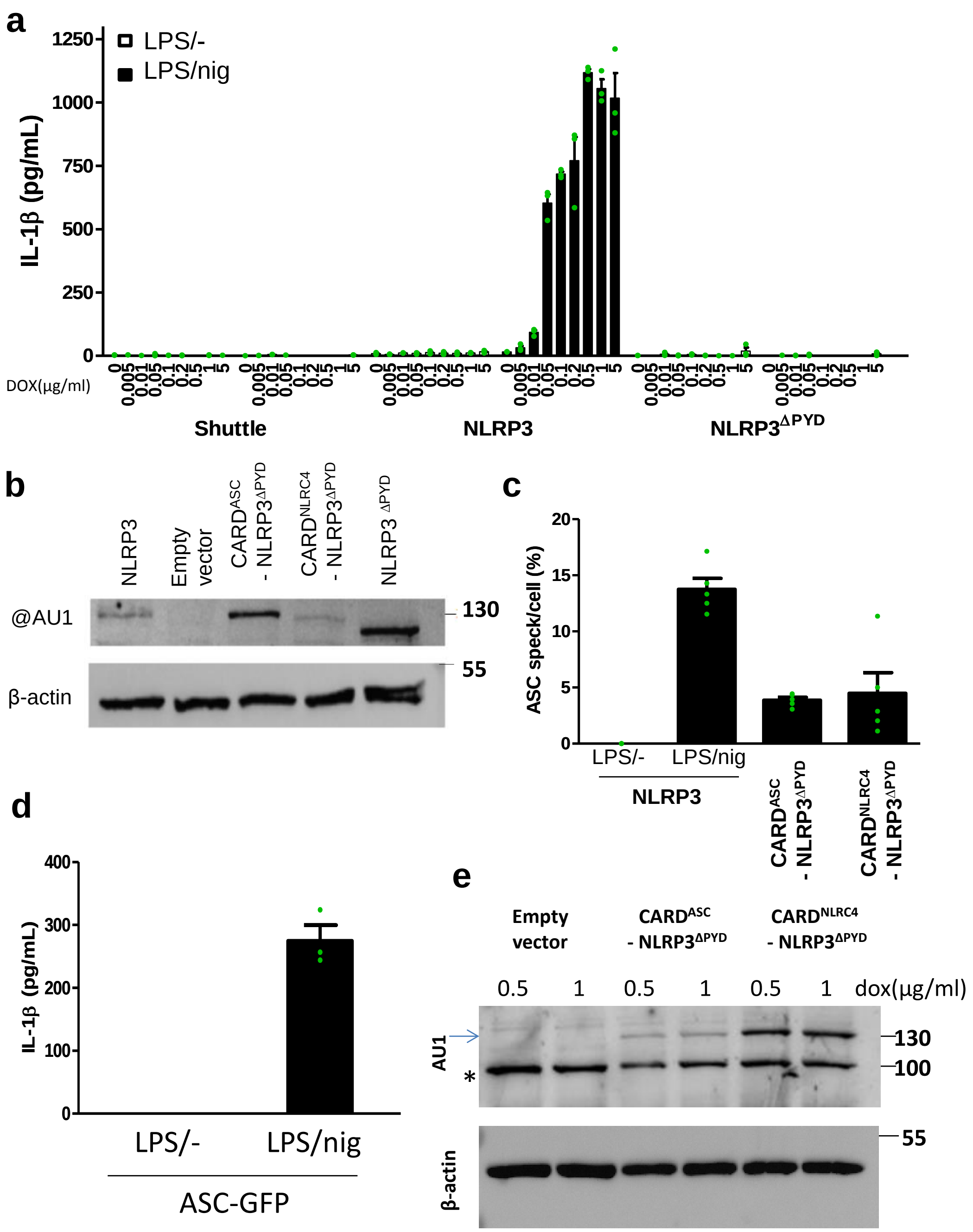

Supplementary Fig. 14. Expression of chimeras in NLRP3$^{-/-}$ and ASC$^{-/-}$ iBMDMs. **a** NLRP3$^{\Delta PYD}$ does not respond to nigericin even at very high doxycycline concentrations. **b** Expression of NLRP3 variants in NLRP3$^{-/-}$ iBMDMs. To facilitate detection these constructs harbor AU1 tag at their C-termini. **c** Analysis of ASC speck formation. Five random frames were used for analysis in each case. **d** Transduction of ASC-GFP construct with constitutive expression into ASC$^{-/-}$ reconstitutes inflammasome activation (the response of LPS-treated cells was below detection limit). **e** Expression of chimeric proteins in ASC$^{-/-}$ iBMDMs. Representative of 2 experiments is shown (a-d). The mean and the s.e.m. of 3 (**a**) or 5 (**c**) biological replicates are shown.

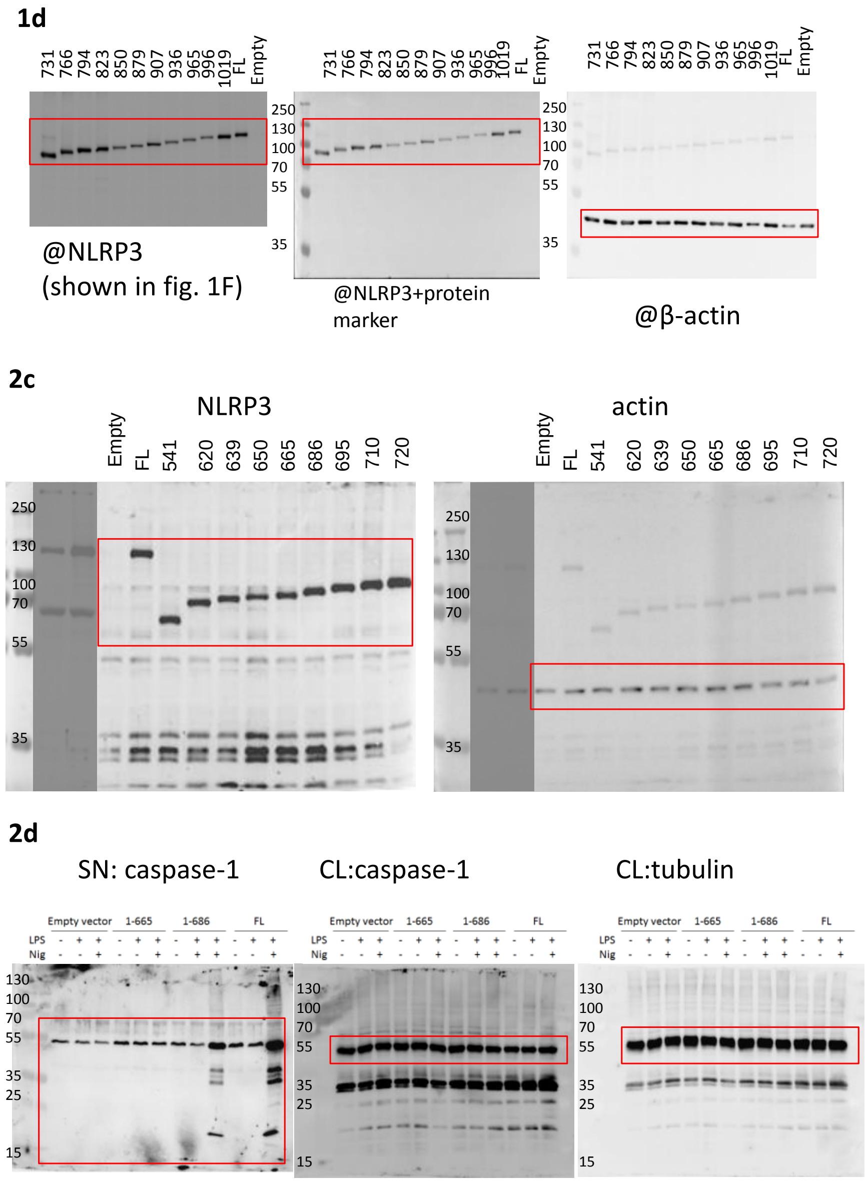

Supplementary Fig. 15. Uncropped Western blots for generating Figures 1d, 2d, 2d. **1d** membrane was first probed for NLRP3, and afterwards for actin. **2c** Membrane was first probed for NLRP3, and afterwards for actin. **2d** Membrane with cell lysates was first probed for caspase-1, the membrane was stripped and afterwards probed for tubulin. Approximate parts shown in cropped versions are outlined in red. The parts that are transparently covered are not shown in the present study.

3b

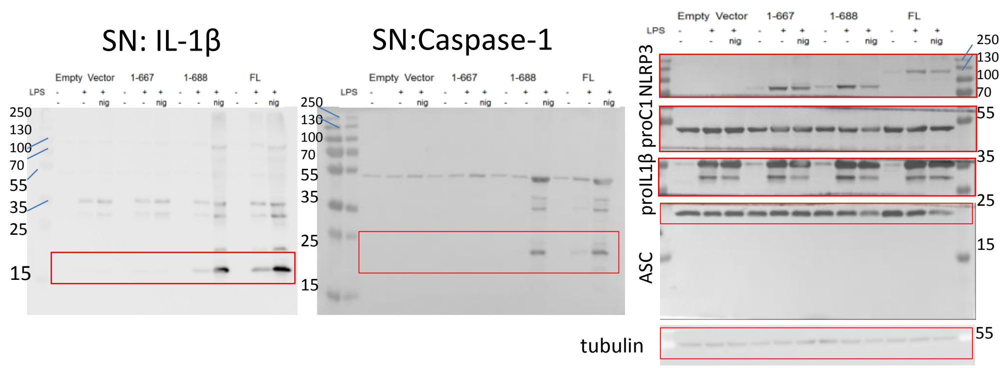

4c

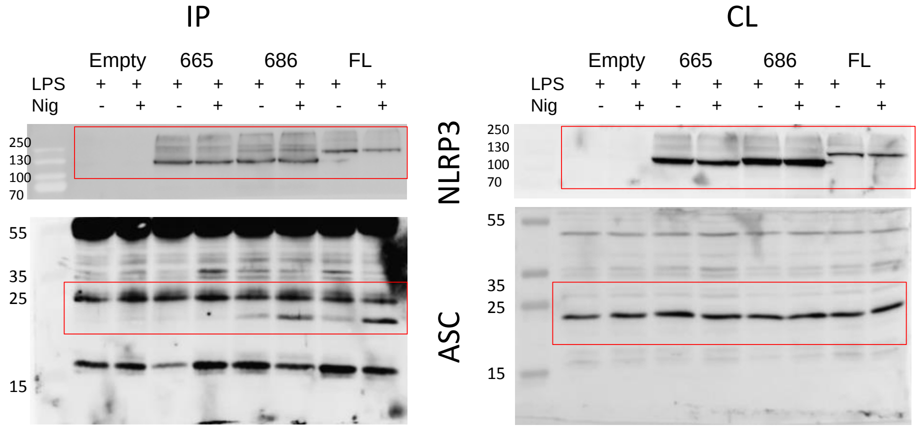

5c

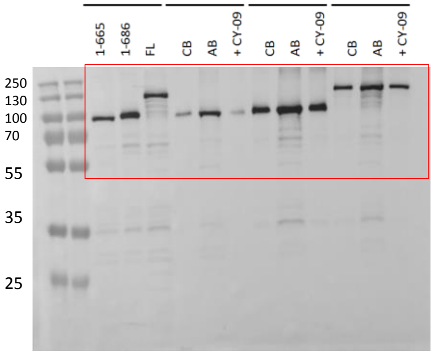

6a

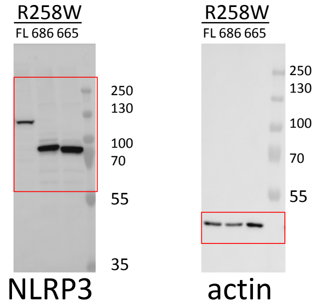

Supplementary Fig. 16. Uncropped Western blots for generating Figures 3b, 4c, 5c, 6a. **3b** The membrane with cell lysate samples was cut horizontally according to protein weight markers and the parts were probed for different proteins, the part that was first probed for caspase-1, was stripped and afterwards probed for tubulin. **4c** Membranes with cell lysate and IP were cut horizontally according to protein weight markers and blot parts were labeled for NLRP3 or ASC. **6a** Samples for NLRP3 and actin analysis were loaded on two different gels.

6b and Suppl. Fig. 6a

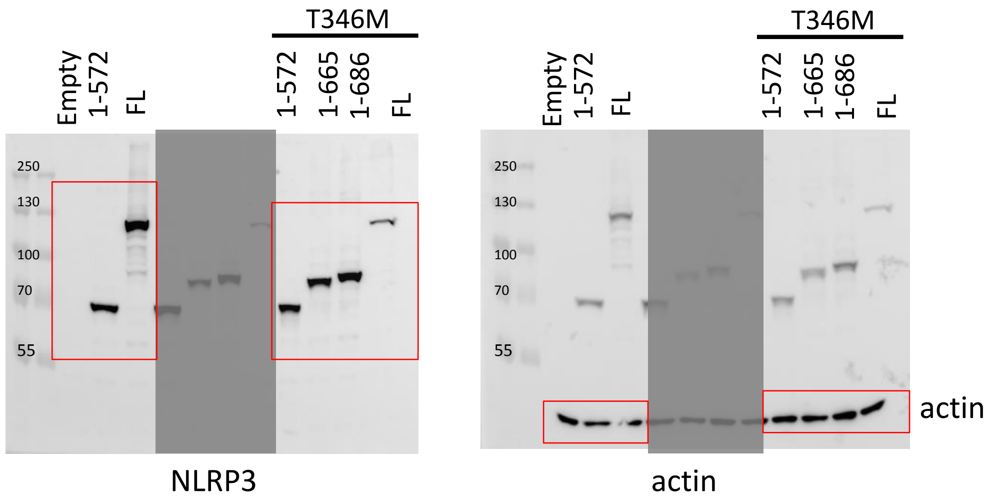

8a-d (AU1)

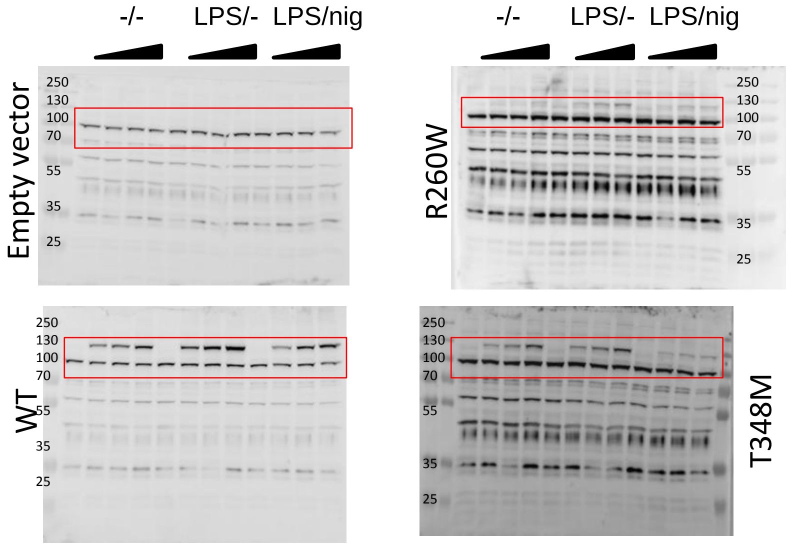

Supplementary Fig. 17. Uncropped blots for generating Fig. 6b, Supplementary Fig. 6a, Figs. 8a-d. The parts that are transparently covered are not shown in the present study.

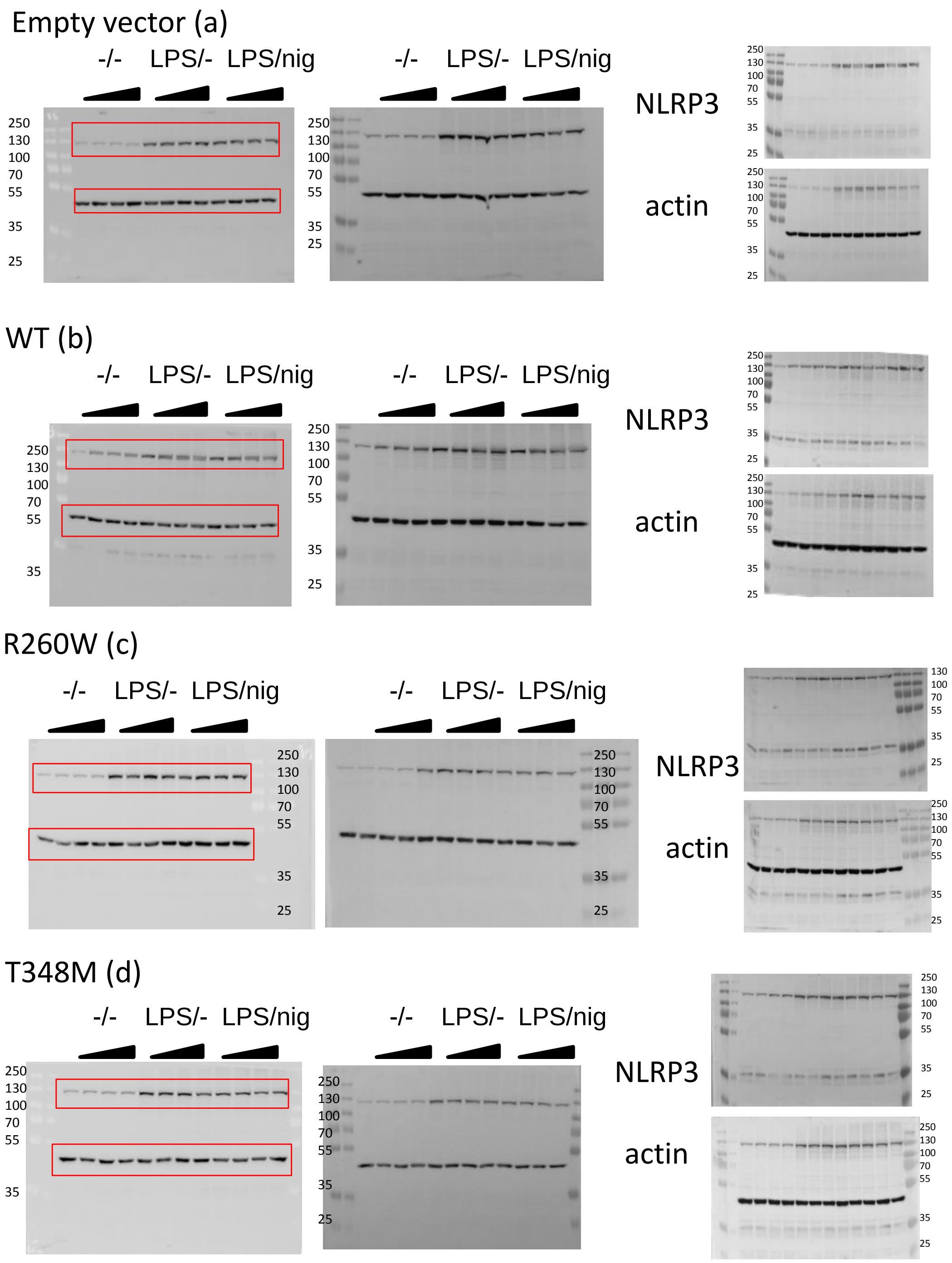

Supplementary Fig. 18. Uncropped blots from Figures 8a-d (NLRP3 & actin). To enable protein quantification, the experiment was performed in 3 biological replicates (the first 2 membranes for each variant were simultaneously probed for NLRP3 and actin, while the third parallel was first probed for NLRP3 and afterwards for actin). Note that protein quantification was performed on luminescence images (dark). Composite images are presented to show the protein marker.

Suppl. Fig. 2d

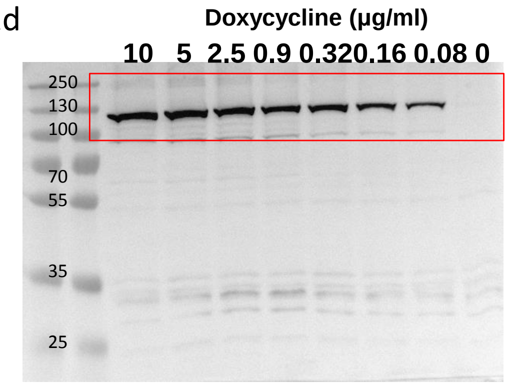

Suppl. Fig. 4b-d

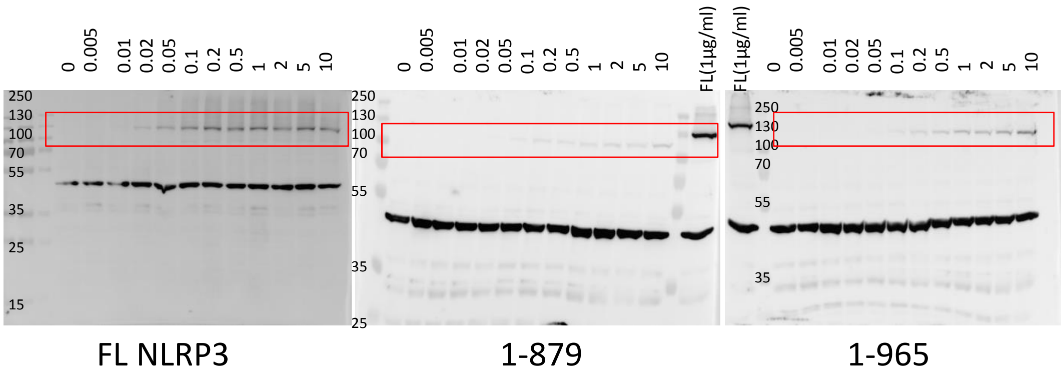

Suppl. Fig. 5a

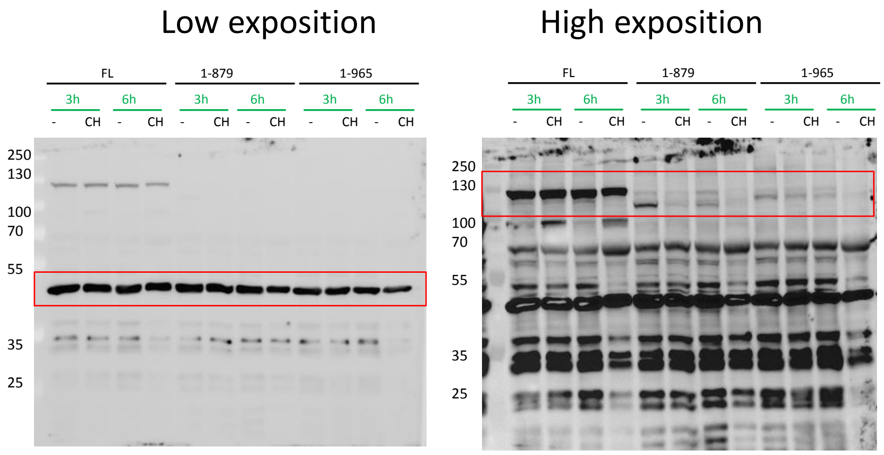

Supplementary Fig. 19. Uncropped blots from Supplementary Figs. 2d, 4b-d, 5a. **Supplementary Fig. 4b-d** membranes were simultaneously probed for NLRP3 and actin. Note that lower exposition images for actin are shown in the main figures, which were also used for quantification. **Supplementary Fig. 5a** membranes were simultaneously probed for NLRP3 and actin.

Suppl. Fig. 5b

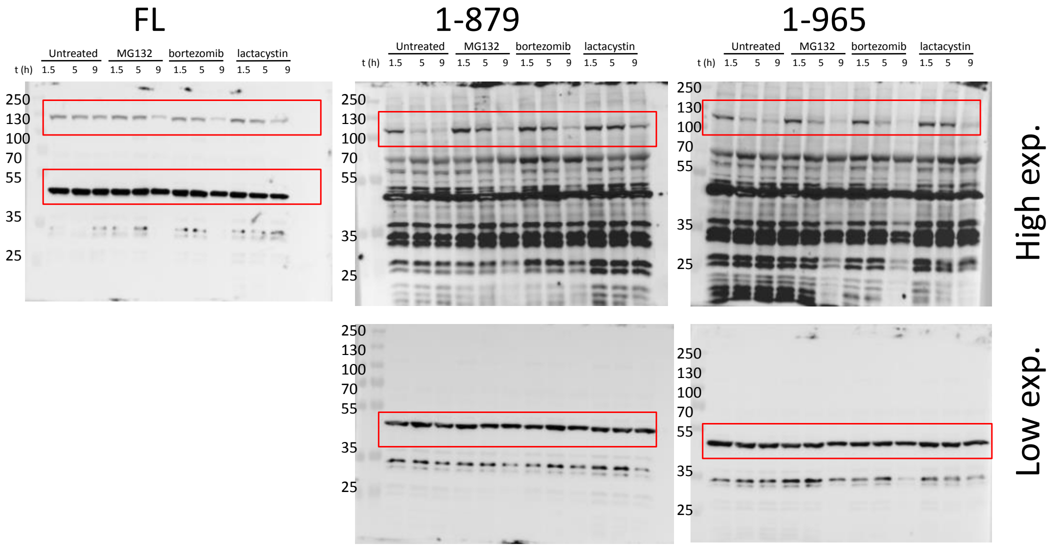

Suppl. Fig. 6b

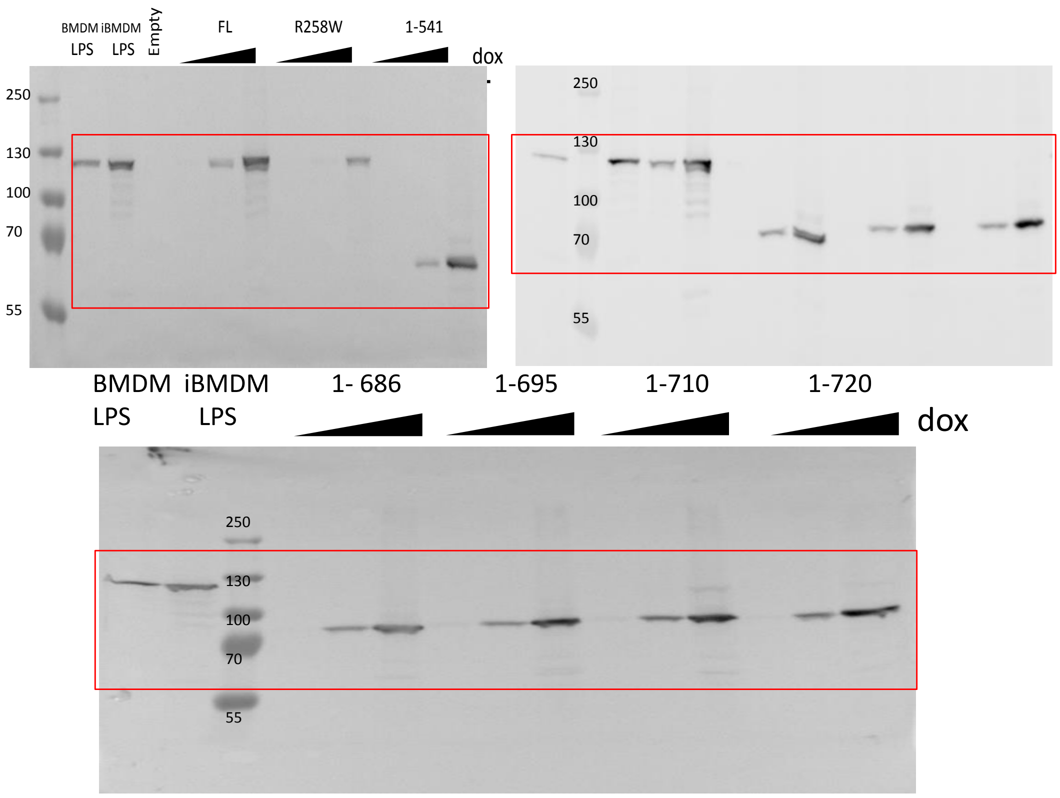

Supplementary Fig. 20. Uncropped blots from Supplementary Figures 5b, 6b. **Supplementary Fig. 5b** membranes were simultaneously probed for NLRP3 and actin.

Suppl. Figure 8d

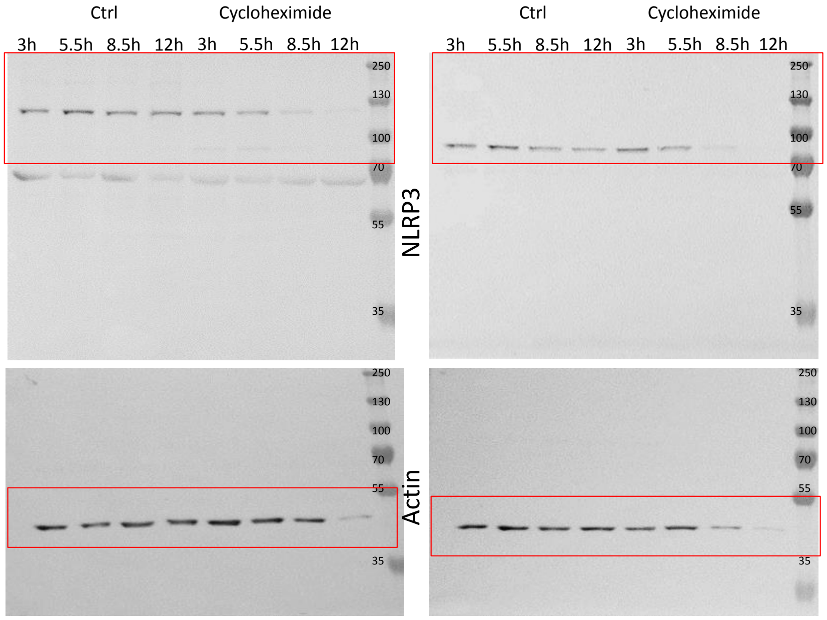

Suppl. Figure 8e

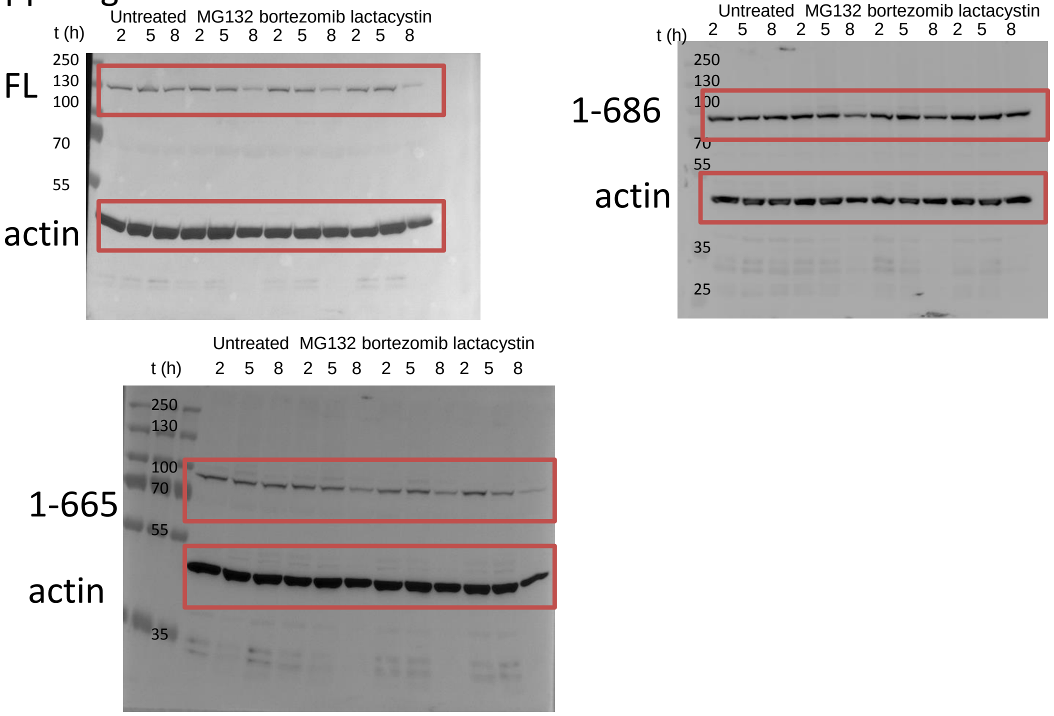

Supplementary Fig. 21. Uncropped blots from Supplementary Figures 8d, 8e. **Supplementary Fig. 8d** blots were first probed for NLRP3, and afterwards for actin, while in **Supplementary Fig. 8e**, membranes were simultaneously probed for NLRP3 and actin.

Suppl. Fig. 9a

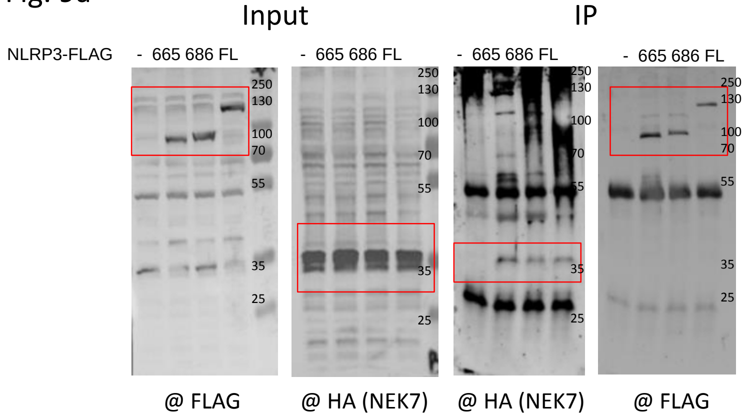

Suppl. Fig. 9b

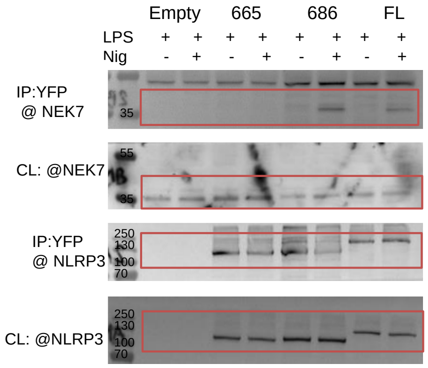

Suppl. Fig.12b

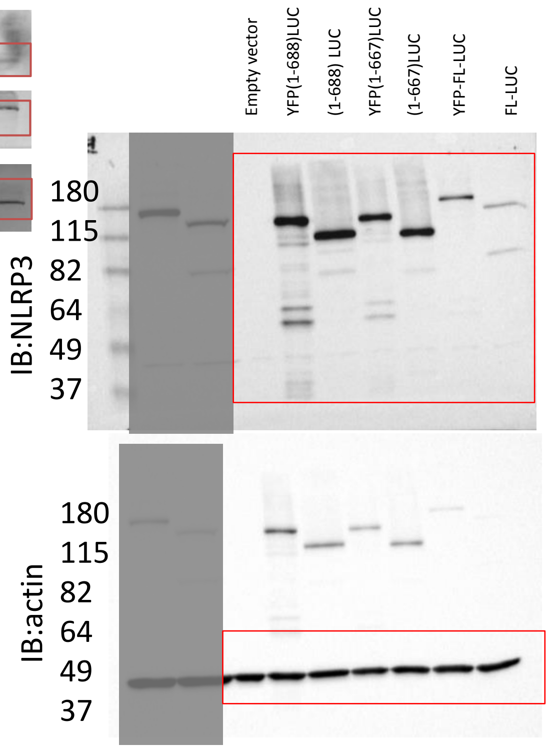

Supplementary Fig. 22. Uncropped blots from Supplementary Figs. 9a, 9b, 12b. **Supplementary Fig. 9b** membranes with cell lysate and IP were cut horizontally according to protein weight markers and blot parts were labeled for NLRP3 or NEK7. **Supplementary Fig. 12b** The parts that are transparently covered are not shown in the present study.

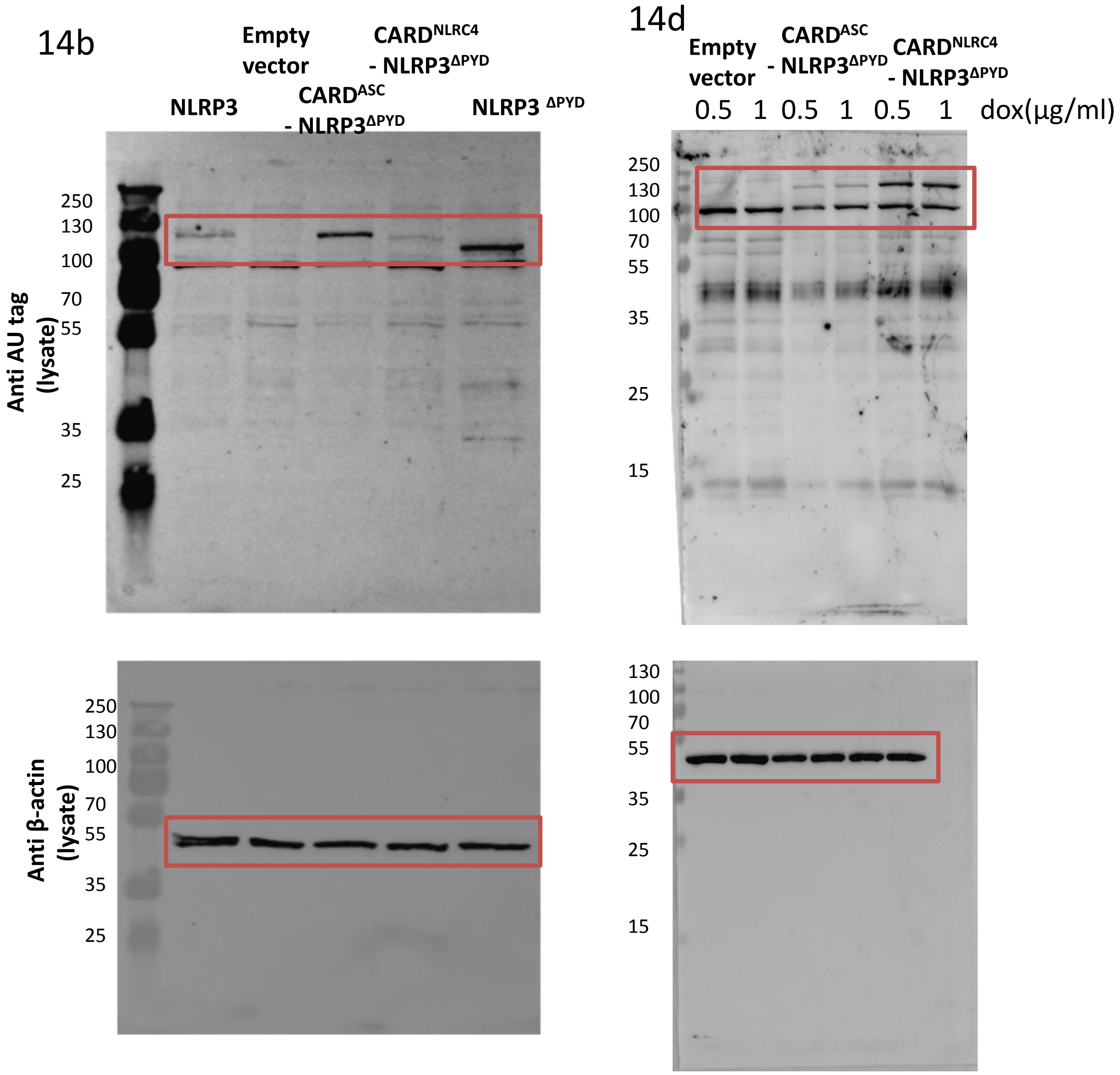

Supplementary Fig. 23: Uncropped blots from Supplementary Figures 14 b, d.

5d

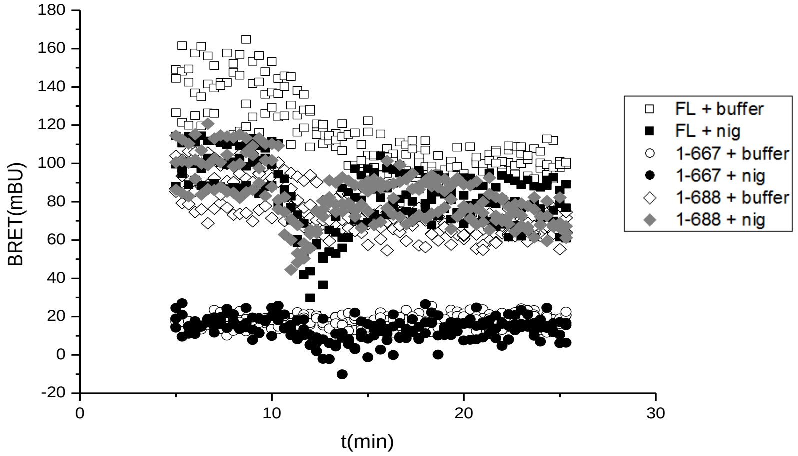

Suppl. Fig. 12

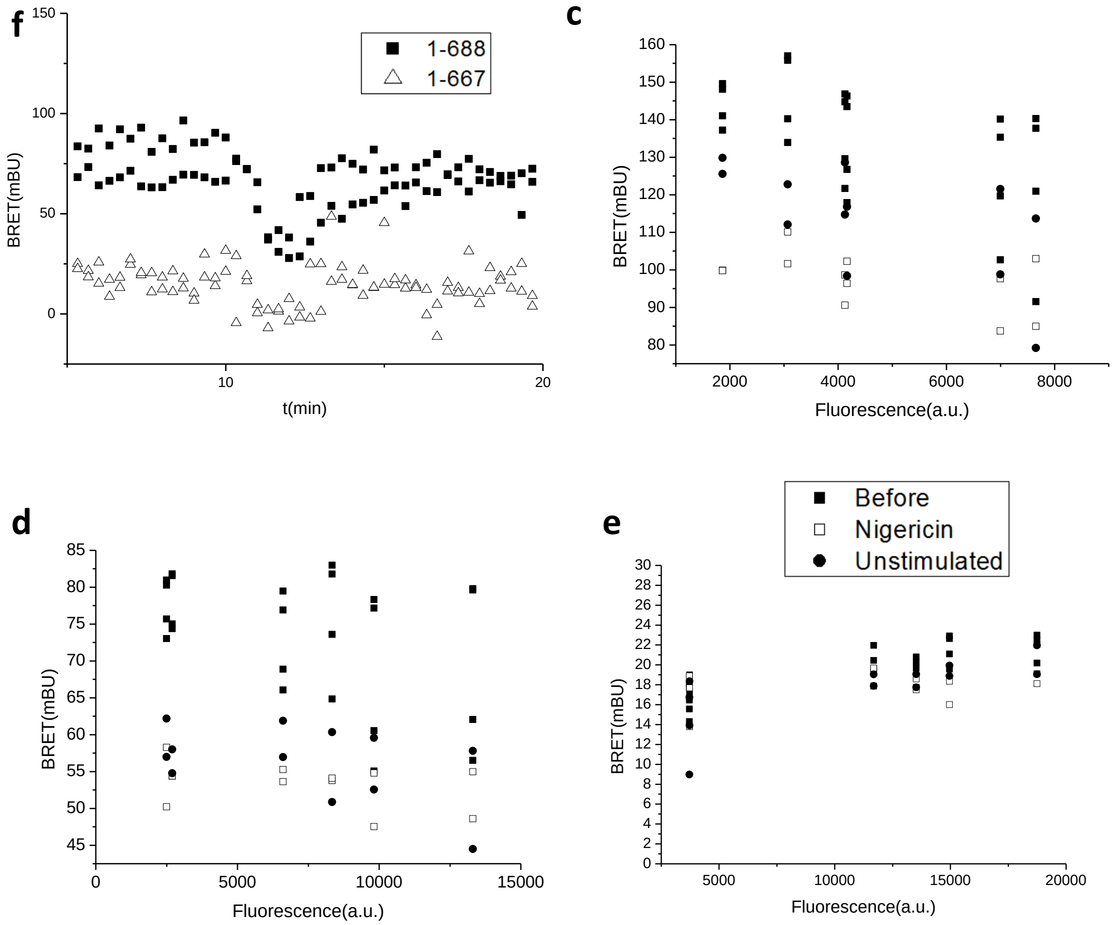

Supplementary Fig. 24. All data points are depicted for charts from Figure 5d and Supplementary Figures 12c-f.

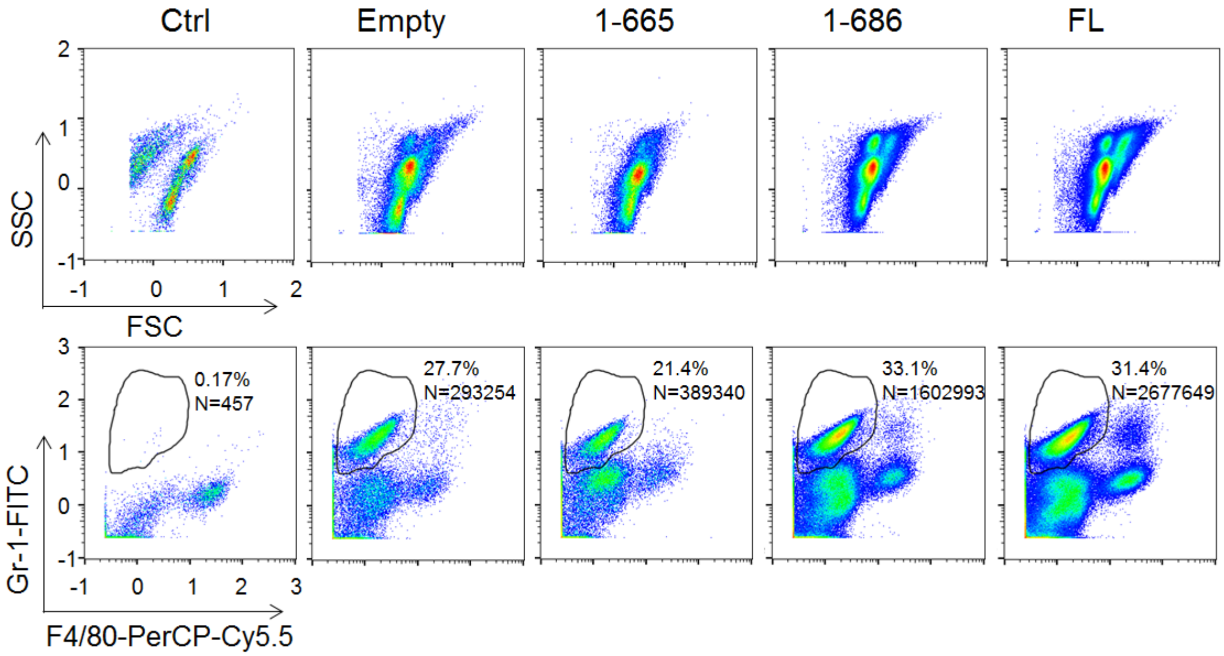

Supplementary Fig. 25. Flow cytometry analysis of peritoneal lavage cells. Peritoneal cells were labeled with Gr-1-FITC and F4/80 PerCP-Cy5.5 and the number of total cells/volume was determined. The total number of Gr-1$^+$/F4/80$^-$ cells (N) was calculated by multiplying the gated percentage and the total number of cells. Representative SSC/FSC (above) and corresponding FL1/FL3 plots (below) are shown (log scale is used).The whole cell population was included in analysis.

Supplementary Table 1: Materials, chemicals and supplies used in the study

MATERIALS	PRODUCER (CAT. NUMBER)
Anti-ASC antibody: HASC-71	Biolegend (653902) (1:500 for IF/microscopy)
Anti-ASC antibody: AL177	Adipogen(AG-25B-0006) (1:1000 for WB)
Anti-mouse caspase-1 p20: Casper-1	Adipogen (AG-20B-0042-C100) (1:1000 for WB)
Anti IL-1β rabbit IgG	Genetex(1G-GTX74034-100) (1:2000 for WB)
Anti-NLRP3: Cryo-2	Adipogen (AG-20B-0014-C100) (1:2000 for WB)
Anti-Flag	Sigma (F7425) (1:320 for WB; 2 μL per IP sample)
Anti-HA	Invivogen (Ab-hatag) (1:667 for WB)
Anti-AU1	Abcam(ab3401) (1:1000 for WB)
anti-green fluorescent protein, rabbit IgG fraction	Invitrogen (A11122) (5 μg/IP sample)
Anti-NEK7	Abcam (ab133514, EPR4900) (1:2000 for WB)
Goat Anti-Mouse IgG (H+L)-HRP	Jackson Immunoresearch (115-035-003) (1:3000 for WB)
Goat polyclonal to rabbit IgG (HRP)	Abcam (ab6721) (1:3000 for WB)
Amersham ECL Mouse IgG, HRP-linked whole Ab (from sheep)	GE Healthcare (NA931) (1:1000 for WB)
Anti-β-actin (C4)-HRP	Santa Cruz (Sc-47778) (1:1000 for WB)
β-Actin (8H10D10) Mouse mAb	Cell Signaling Technology (#3700) (1: 5000 for WB)
α/β-Tubulin Antibody	Cell Signaling Technology (#2148) (1:4000 for WB)
Anti-Mouse Ly-6G (Gr-1) FITC, clone: RB6-8C5	eBioscience/ Thermo Fisher Scientific (11-5931-82) (1.5 μL/FC sample)
Anti-Mouse F4/80 Antigen PerCP-Cy5.5, clone BM8	eBioscience/ Thermo Fisher Scientific (45-4801-82) (1.5 μL/FC sample)
Donkey anti-Mouse IgG (H+L) Highly Cross-Adsorbed Secondary Antibody, Alexa Fluor 488	Invitrogen/ Thermo Fisher Scientific (A21202) (1:200 for IF/microscopy)
Protein A-HRP	Abcam (ab7456) (1:1000 for WB)
Ultrapure LPS e. coli O111:B4	Invivogen (tlrl-3pelps), Adipogen (IAX-100-012-M001)
Pam2CSK4	Invivogen (tlrl-pm2s-1)
lactacystin	Sigma (L6785)
bortezomib	Santa Cruz (sc-217785)
MG-132	Sigma (C2211)
BenchMark Pre-stained Protein standard	Thermo Fisher Scientific (10748010)
PageRuler Plus Prestained Protein Ladder	Thermo Fisher Scientific (26619)
ATP Separopore 4B-CL	bioWorld (20181080-1)
Separopore 4B-CL	bioWorld (20181032-1)
CY-09	MedChemExpress (HY-103666)
G5	Med Chem Express(HY-100738)
shikonin	Enzo (BML-CT-115)
MCC950	Avistron (AV02509)
Nigericin	Sigma (N7143)
Silica	Invivogen (tlrl-sio)
Imject Alum	Thermo Fisher Scientific (77161)
Imiquimod	Invivogen (tlrl-imqs)
Cycloheximide	Sigma (C7698)
Phusion High-Fidelity DNA Polymerase	Thermo Fisher Scientific (F530L)

BamHI	Thermo Fisher Scientific (ER0055)
EcoRI	Thermo Fisher Scientific (ER0275)
NotI	Thermo Fisher Scientific (ER0592)
Coelenterazine-H	Invitrogen/Thermo Fisher Scientific (C6780)
Adenosine 5′-triphosphate disodium salt hydrate	Sigma (A2383)
saponin	Sigma (84510)
Phalloidin-CF647	Santa Cruz Biotechnology (sc-363797)
Paraformaldehyde 32% solution	Electron Microscopy Sciences (15714-S)
DMEM + GlutaMAX-I	Gibco/ Thermo Fisher Scientific (21885-025)
Fetal bovine serum heat inactivated	Gibco/ Thermo Fisher Scientific (10500-064)
Poly-L-lysine solution	Sigma-Aldrich (P8920)
Lipofectamine 2000 Transfection reagent	Invitrogen /Thermo Fisher Scientific (11668019)
Mouse M-CSF	eBioscience /Thermo Fisher Scientific (14-8983-62)
Complete, mini protease inhibitor cocktail tablets	Roche (Sigma-Aldrich) (4693124001)
Rotihistofix	Roth (P087.5)
Hoechst	ImmunoChemistry Technologies (639)
puromycin	Invivogen (ant-pr-1)
G418 solution	Sigma-Aldrich (G8168)
Prolong Diamond Antifade solution with DAPI	Thermo Fisher Scientific (P36966)
Z-VAD-FMK	Invivogen (tlrl-vad)
Mouse IL-1β ELISA Kit	eBioscience/ThermoFisherScientific (88-7013-86)
Mouse IL-18 ELISA Kit	eBioscience/ThermoFisherScientific(BMS618/3)
Mouse TNF ELISA	ebioscience/ThermoFisherScientific (5017331)
Mouse IL-6	ebioscience/ThermoFisherScientific (88-7064-88)
Cytotoxicity Detection Kit (LDH)	Roche (11644793001)
poly-L-lysine-coated slide	Corning (354085)
μ-slide 8 well	Ibidi (80826)
Amicon Ultra Centrifugal Filters 10 kDa	Merck Millipore (UFC5010)

Supplementary Table 2: Oligonucleotides used for construct preparation

OLIGONUCLEOTIDES	SEQUENCE
O1: Nlrp3-BamHI-F	CGGGATCCGCCACCATGAC
O2: Nlrp3-(1-541)-R	GGAATTCCTTACACTGTCTCGCCTTCGG
O3: Nlrp3-(1-572)-R	GGAATTCCTTAGATCAGGTATCCCTTCTCGAACT
O4: Nlrp3-(1-620)-R	GGAATTCCTTACTGGAGTTTTTTAGCCTTAGC
O5: Nlrp3-(1-639)-R	GGAATTCCTTAATCCTCTTCCTGCATCTCATA
O6: Nlrp3-(1-650)-R	GGAATTCCTTACTTAGGGAAGTGATCCATAGC
O7: Nlrp3-(1-665)-R	GGAATTCCTTAAGATGACACCACGTGATCC
O8: Nlrp3-(1-686)-R	GGAATTCCTTAGGGGCTGTTGTGGAAGAA
O9: Nlrp3-(1-695)-R	GGAATTCCTTAGCCCCTGCGTTCCTCTT
O10: Nlrp3-(1-710)-R	GGAATTCCTTAGTGGGTGTCGGGGAACA
O11: Nlrp3-(1-720)-R	GGAATTCCTTAACAGCAGTTGACCAGCCTG
O12: Nlrp3-(1-731)-R	GGAATTCCTTAGAACAGGCCTCTGCAGAAG
O13: Nlrp3-(1-766)-R	GGAATTCCTTATCCGGGATGCTGCAGAG
O14: Nlrp3-(1-794)-R	GGAATTCCTTAAGAGCTGCTCAGCACGC
O15: Nlrp3-(1-823)-R	GGAATTCCTTACAGGAGATGTTTCAGGCCCA
O16: Nlrp3-(1-850)-R	GGAATTCCTTAGCTGGACAGCACCAGG
O17: Nlrp3-(1-879)-R	GGAATTCCTTAGGGGTCCTTCATTTTCTCG
O18: Nlrp3-(1-907)-R	GGAATTCCTTATGTCTTGAGCACGCTGGTC
O19: Nlrp3-(1-936)-R	GGAATTCCTTAGGGGTGCAGCAGGCCT
O20: Nlrp3-(1-965)-R	GGAATTCCTTATGTCAGGATGGTAGACAGGTT
O21: Nlrp3-(1-996)-R	GGAATTCCTTACAGACATCCCTGCTGCTTC
O22: Nlrp3-(1-1019)-R	GGAATTCCTTACTGCAGGGCTTCCAGG
O23: F-hNLRP3	CGGGATCCGCCACCATGAAGATGGCAAGCACCC
O24: R-hNLRP3(FL)	ATTTGCGGCCGCCTACCAAGAAGGCTCAAAGACGAC
O25: R-hNLRP3(1-667)	ATTTGCGGCCGCCTAGGAAGAAACCATGTGGTCCATTC
O26: R-hNLRP3(1-688)	ATTTGCGGCCGCCTAGGGCATGTTATGGAGAAACCC
O28: F-flag	CGGGATCCGCCACCATGGATTACAAAGACGATGACGATAAAG
O29: R-luc	ATTTGCGGCCGCTTACTGCTCGTTCTTCAGCACTC
O30: R-1-667-intraBRET	CACCTTGCTGGTAACCGGTAAGGAAGAAACCATGTGGTCCATTC
O31: F-1-667-intraBRET	GAATGGACCACATGGTTTCTTCCTTACCGGTTACCAGCAAGGTG
O32: R-1-688-intraBRET	CACCTTGCTGGTAACCGGTAAGGGCATGTTATGGAGAAACC
O33: F-1-688-intraBRET	GGTTTCTCCATAACATGCCCTTACCGGTTACCAGCAAGGTG
O34: BamHI-kozak-ATG-cardNLRC4	CGGGATCCGCCACCATGAATTTCATAAAGGACAATAGCCGA
O35: R-NLRP3deltaPYD-cardNLRC4	GGTGCAGGTATCGTTCCATGTCTGATGAAAAAGACTTTGTCCATTC
O36: F-cardNLRC4-NLRP3deltaPYD	AATGGACAAAGTCTTTTTCATCAGACATGGAACGATACCTGCACCA
O37: R-Nlrp3deltaPYD-stop-EcoRI	CCGAATTCTCAGATGTACCGGTAGG
O38: F-BamHI-kozak-ATG-cardASC	GGATCCGCCACCATGGCAGCCAAGCCAGGCCTG
O39: R-NLRP3deltaPYD-linker-cardASC	GCTGGTGCAGGTATCGTTCCACCCCCCTGATCCTCCGCTCCGCTCCAGGTCC
O40: f-cardASC-linker-NLRP3deltaPYD	AGGACCTGGAGCGGAGCGGAGGATCAGGGGGGTGGAACGATACCTGCACCAGC

O41: F-BamHI-kozak-ATG-linkercardASC	GGATCCGCCACCATGATCCAGGCCCCTCCTCAGTC
O42: Flag-CARD^{NLRC4}-R	CCGAATTCTCACTTGTCATCGTCATCCTTGTAATCTGTCTGATGAAAAAGACTTTGTCCA
O43: CARD^{ASC}-F	CGGGATCCGCCACCATGG
O44: Flag-CARD^{ASC}-R	TAAAGCGGCCGCTTACTTGTCATCGTCATCCTTGTAATCCCCCCCTGATCCTCCG
O45: C6A-F	GACCTCCGTGCGGGCCAAGCTCGCCCAG
O46: C6A-R	CTGGGCGAGCTTGGCCCGCACGGAGGTC
O47: C104A-F	CAGCAGCATGGTCGCCCAGGAAGATAGTC
O48: C104A-R	GACTATCTTCCTGGGCGACCATGCTGCTG
O49: C667A-F	GGTGTCATCTTTCGCCATCAAGAACTGC
O50: C667A-R	GCAGTTCTTGATGGCGAAAGATGACACC
O51: C671A-F	CTGCATCAAGAACGCCCACAGAGTCAAG
O52: C671A-R	CTTGACTCTGTGGGCGTTCTTGATGCAG
O53: C667A C671A-F	GGTGTCATCTTTCGCCATCAAGAACGCCCACAGAGTCAAG
O54: C667A C671A-R	CTTGACTCTGTGGGCGTTCTTGATGGCGAAAGATGACACC
O55: H672A R673A-F	CATCAAGAACTGCGCCGCAGTCAAGACCCTG
O56: H672A R673A-R	CTGGGCGAGCTTGGCCCGCACGGAGGTC
O57: R258W-F	CTTCATCCATTGCTGGGAAGTGTCCC
O58: R258W-R	GGGACACTTCCCAGCAATGGATGAAG
O59: T346M-F	CTGCTGATCACCATGAGACCCGTGGCC
O60: T346M-R	GGCCACGGGTCTCATGGTGATCAGCAG
O61: NLRP3dPYD-F	CGGGATCCGCCACCATGTGGAACGATACCTGCACCA

SUPPLEMENTARY REFERENCES

1. The UniProt, C. UniProt: the universal protein knowledgebase. *Nucleic Acids Res* **45**, D158-D169 (2017).

2. Bej, A., et al. LRRsearch: An asynchronous server-based application for the prediction of leucine-rich repeat motifs and an integrative database of NOD-like receptors. *Comput Biol Med* **53**, 164-170 (2014).

3. Albrecht, M., Domingues, F. S., Schreiber, S., Lengauer, T. Structural localization of disease-associated sequence variations in the NACHT and LRR domains of PYPAF1 and NOD2. *FEBS Lett* **554**, 520-528 (2003).

4. Proell, M., Riedl, S. J., Fritz, J. H., Rojas, A. M., Schwarzenbacher, R. The Nod-like receptor (NLR) family: a tale of similarities and differences. *PLoS One* **3**, e2119 (2008).

5. Hu, Z., et al. Crystal structure of NLRC4 reveals its autoinhibition mechanism. *Science* **341**, 172-175 (2013).

6. Maekawa, S., Ohto, U., Shibata, T., Miyake, K., Shimizu, T. Crystal structure of NOD2 and its implications in human disease. *Nature communications* **7**, 11813 (2016).

7. Yang, J., et al. The I-TASSER Suite: protein structure and function prediction. *Nature methods* **12**, 7-8 (2015).
